# iPSCs derived from esophageal atresia patients reveal SOX2 dysregulation at the anterior foregut stage

**DOI:** 10.1242/dmm.049541

**Published:** 2022-11-28

**Authors:** Suleen Raad, Anu David, Melanie Sagniez, Bastien Paré, Zakaria Orfi, Nicolas A. Dumont, Martin A. Smith, Christophe Faure

**Affiliations:** ^1^ Esophageal Development and Engineering Laboratory, CHU Sainte-Justine Research Center, 3175 Côte Sainte-Catherine, Montréal, Quebec H3T 1C5, Canada; ^2^ CHU Sainte-Justine Research Center, 3175 Côte Sainte-Catherine, Montréal, Quebec H3T 1C5, Canada; ^3^ Department of Biochemistry and Molecular Medicine, Faculty of Medicine, University of Montreal, Montréal, Quebec H3T 1J4, Canada; ^4^ School of Rehabilitation, Faculty of Medicine, Université de Montréal, Montréal, Quebec H3T 1J4, Canada; ^5^ Esophageal Atresia Clinic and Division of Pediatric Gastroenterology Hepatology and Nutrition, CHU Sainte-Justine, 3715 Côte Sainte-Catherine, Université de Montréal, Montréal, Quebec H3T1C5, Canada

**Keywords:** Esophageal atresia/tracheoesophageal fistula, iPSCs, Esophageal organoids, Anterior foregut

## Abstract

A series of well-regulated cellular and molecular events result in the compartmentalization of the anterior foregut into the esophagus and trachea. Disruption of the compartmentalization process leads to esophageal atresia/tracheoesophageal fistula (EA/TEF). The cause of EA/TEF remains largely unknown. Therefore, to mimic the early development of the esophagus and trachea, we differentiated induced pluripotent stem cells (iPSCs) from EA/TEF patients, and iPSCs and embryonic stem cells from healthy individuals into mature three-dimensional esophageal organoids. CXCR4, SOX17 and GATA4 expression was similar in both patient-derived and healthy endodermal cells. The expression of the key transcription factor SOX2 was significantly lower in the patient-derived anterior foregut. We also observed an abnormal expression of NKX2.1 (or NKX2-1) in the patient-derived mature esophageal organoids. At the anterior foregut stage, RNA sequencing revealed the critical genes *GSTM1* and *RAB37* to be significantly lower in the patient-derived anterior foregut. We therefore hypothesize that a transient dysregulation of SOX2 and the abnormal expression of NKX2.1 in patient-derived cells could be responsible for the abnormal foregut compartmentalization.

## Teaser

**Summary:** Differentiation of iPSCs derived from patients with esophageal atresia to mature esophageal organoids reveals a dysregulation of SOX2 and NKX2.1, key factors in esophagus and trachea development.

## INTRODUCTION

The esophagus and trachea originate from the endodermal diverticulum in the anterior foregut tube. Well-regulated and organized cellular and molecular events result in the separation of the anterior foregut tube into the esophagus and trachea (Raad et al., 2020; Billmyre et al., 2015). Disruption of the compartmentalization process results in severe esophageal congenital anomalies, such as esophageal atresia with or without tracheoesophageal fistula (EA/TEF), affecting one in 3000 newborns (van Lennep et al., 2019). Several types of EA/TEF have been described based on the location of the malformation and the affected structures, with the most common being type C (>80% of cases), in which the upper segment of the esophagus ends in a blind pouch, and a fistula connects the lower part to the trachea. Other less common subtypes include type A (8-10% of cases), in which no fistula exists, but the esophagus is disconnected (Clark, 1999). EA/TEF-associated anomalies (cardiac, anal, renal, limb or vertebral) are also reported in 30-50% of syndromic cases. Monogenetic causes account for a minority of EA/TEF cases (<5%), most often in syndromic cases, such as anophthalmia-esophageal-genital (AEG) syndrome (*SOX2* mutations), Feingold syndrome (*MYCN* mutations), CHARGE syndrome (*CHD7* mutations), Pallister–Hall syndrome (*GLI3* mutations) and mandibulofacial dysostosis (*EFTUD2* mutations) (Stoll et al., 2009). Studies have also shown a multigenic architecture of rare variants in several genes, which discriminate EA/TEF cases from controls (Wang et al., 2021). However, the cause of EA/TEF remains largely unknown, and rare genetic variants are seldom reported in non-syndromic, isolated cases. EA/TEF is thus considered a multifactorial anomaly resulting from genetic and environmental factors (Brosens et al., 2014).

During embryogenesis, the esophagus and trachea arise after the separation of the anterior foregut endoderm common tube at weeks 4-5 in humans and embryonic days 9.5-11.5 in mice. In animal models (mouse and *Xenopus*), the dorsal/ventral patterning of the anterior foregut allows spatial specification of the two presumptive organs: the esophagus on the dorsal side of the anterior foregut tube (characterized by the expression of the transcription factor SOX2) and the trachea on the ventral side of the foregut tube [characterized by the expression of the *NKX2.1* (or *NKX2-1*) gene] (Minoo et al., 1999; Que et al., 2007; Kim et al., 2019). Studies in mice have also demonstrated that the dorsal/ventral patterning is initiated by gradual expression of mesodermal Wnt2/2b, Bmp4, and noggin along the dorsal-ventral axis. BMP signaling pathway inhibits SOX2 expression on the dorsal side of the anterior foregut (Domyan et al., 2011) and drives Nkx2.1 expression toward the tracheal lineage. Functional genomic studies in mice and *Xenopus* have also been used to mimic the genetics and morphogenetic regulation of normal and abnormal foregut compartmentalization representing human esophageal anomalies, such as EA/TEF (Kim et al., 2019; Fausett and Klingensmith, 2012; Raad et al., 2020). However, these studies utilize methods resulting in functional loss of specific genes that may not represent the genetic complexity observed in humans. The human esophagus differs structurally and morphologically from the mouse esophagus (Rosekrans et al., 2015). Therefore, there is a need to have a representative model of human esophagus development to not only decipher, but also understand the possible mechanisms leading to EA/TEF. Induced pluripotent stem cells (iPSCs) offer an excellent tool in gaining insights not only into human embryonic and developmental ontologies, but also to model diseases (Karagiannis et al., 2019; Rowe and Daley, 2019) through the directed differentiation to specific organs originating from all three germ layers. To date, patient-derived iPSCs have not yet been used to study digestive malformations. Recently, patient-derived iPSCs were used to study congenital heart diseases, in which intrinsic defects were observed in differentiated cardiomyocytes derived from these iPSCs (Miao et al., 2020; Hrstka et al., 2017; Yang et al., 2017). Over the last few years, studies using healthy human iPSCs have been used to generate mature esophageal epithelia (Zhang et al., 2018; Trisno et al., 2018), and confirmed previous findings on the key role of SOX2 in promoting esophageal specification and the critical roles of the BMP, TGFβ and WNT signaling pathways during esophageal development (Que et al., 2006, 2007; Teramoto et al., 2020; Zhang et al., 2018; Trisno et al., 2018; Li et al., 2007; Woo et al., 2011; Domyan et al., 2011).

Therefore, to mimic the normal and abnormal early development of the esophagus and trachea, the objective of this study was to differentiate embryonic stem cells (ESCs) and iPSCs from healthy individuals and iPSCs from EA/TEF type C pediatric patients into mature esophageal organoids in matrix- and xenogeneic-free culture conditions. We adapted and modified a stepwise differentiation protocol (Matsuno et al., 2016; Zhang et al., 2018; Giroux et al., 2017; DeWard et al., 2014) by manipulating key signaling pathways involved in esophagus development. We investigated the gene and protein expression profiles of key signaling molecules in patient cells and compared them to healthy cells at each developmental stage. Furthermore, by combining targeted gene expression and nanopore RNA sequencing, we demonstrated that patient-derived cells exhibit unique molecular signatures, especially at the anterior foregut stage. Our study establishes a basic framework to understand the morphogenesis and mechanisms involved during early esophageal development by using patient-derived iPSCs.

## RESULTS

**Fig. 1.**
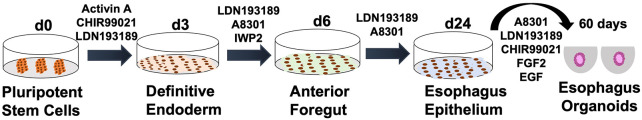
**Stepwise differentiation protocol of human pluripotent stem cells into esophagus organoids.** Illustration of how different signaling pathways can be manipulated to differentiate pluripotent stem cells into each developmental stage, starting from the definitive endoderm, anterior foregut, esophagus epithelium and esophagus organoids. ‘d’ indicates the number of days. Activin A is a dimeric growth and differentiation factor that activates the Nodal/TGFβ pathway. CHIR99021 is an aminopyrimidine derivative that is potent as a GSK3 inhibitor, a key inhibitor of the WNT pathway, and thus CHIR99021 treatment activates the WNT pathway. LDN193189 is a dihydrochloride, a potent and selective ALK2 and ALK3 inhibitor. It inhibits BMP4-mediated Smad1/5/8 activation. A8301 is a potent inhibitor of the TGFβ type I receptor ALK5 kinase, the type I activin/nodal receptor ALK4 and type I nodal receptor ALK7. IWP2 inhibits the WNT pathway at the level of the pathway activator porcupine (PORCN), leading to WNT secretion and signaling capability. FGF2 is a growth factor belonging to the FGF superfamily. It stimulates cell proliferation. EGF is a potent growth factor belonging to the EGF family. It induces cell proliferation, differentiation and survival.

Our modified protocol includes the stepwise differentiation of pluripotent stem cells (PSCs) into mature esophageal organoids with checkpoints at four developmental stages: (1) the definitive endoderm, (2) the anterior foregut, (3) the mature esophageal epithelium and (4) three-dimensional esophageal organoids (Fig. 1). We compared the differentiation potential to these stages among healthy PSCs [embryonic stem cell line H9 (female)], an iPSC-derived from a non-familial healthy male and EA/TEF iPSCs (two males and one female).

### Derivation of EA/TEF patient iPSCs

iPSC lines from three pediatric isolated type C EA/TEF patients (two males and one female) without any associated malformations were established by reprogramming peripheral blood mononuclear cells in the Stem Cell core facility at CHU-Sainte Justine. Their pluripotency was confirmed by the mRNA expression of pluripotent genes *SOX2*, *NANOG* and *OCT4*, and immunofluorescence staining for the proteins SOX2, NANOG and OCT4 and the glycolipid SSEA4. All three iPSC cell lines had a normal karyotype, had no pathogenic genetic variants in established EA/TEF risk genes and showed the ability to differentiate into the three germ layers as evidenced through teratoma formation (Raad et al., 2022).

### Similar differentiation potential of healthy and EA/TEF patient PSCs into definitive endodermal cells

**Fig. 2.**
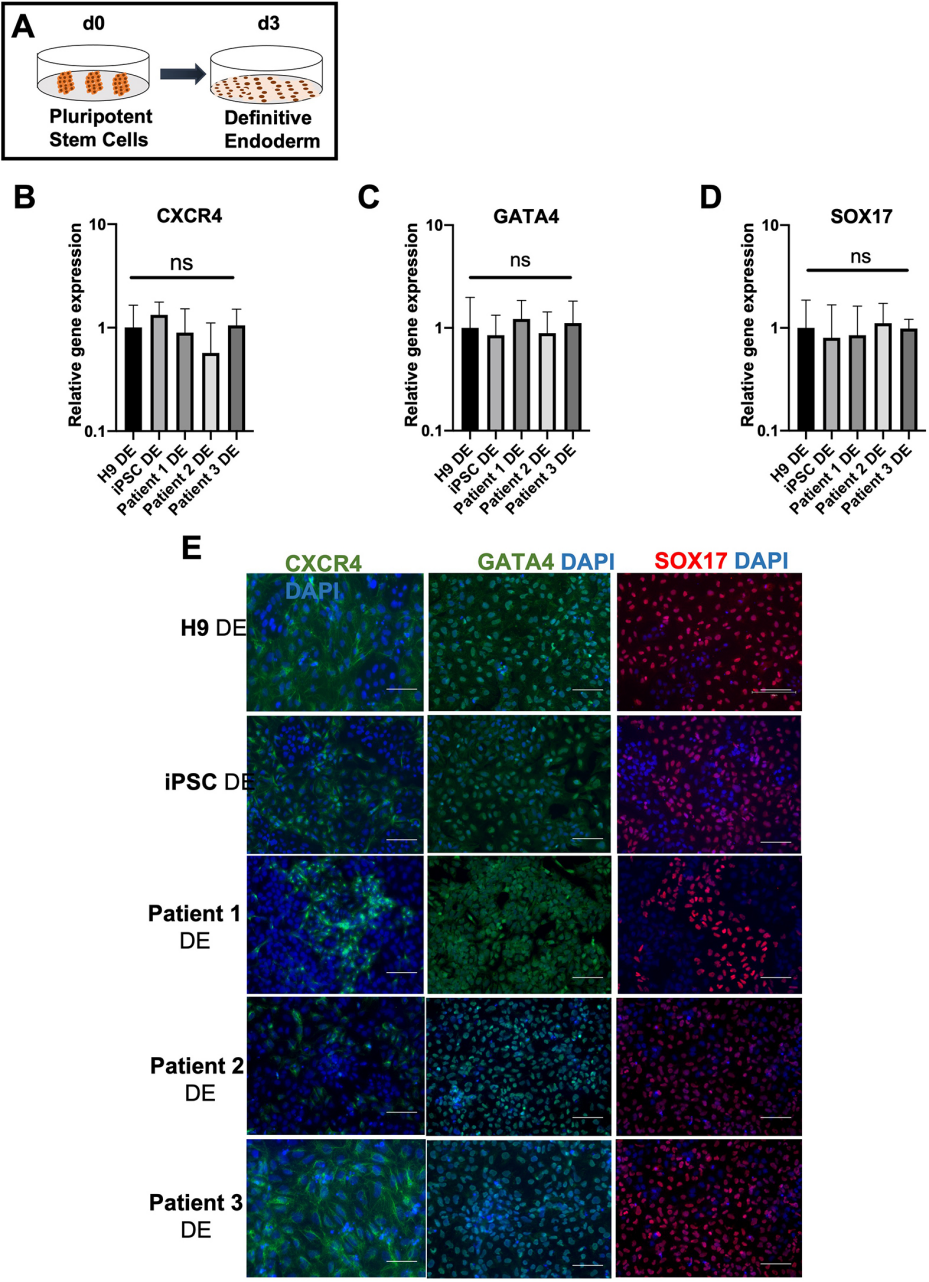
**Differentiation of healthy and EA/TEF patient-derived pluripotent stem cells into the definitive endoderm.** (A) Illustration of the first stage of differentiation into the definitive endoderm (DE). (B-D) Expression of the key endodermal markers *CXCR4*, *GATA4* and *SOX17* was quantified by RT-qPCR and reported as fold change. The fold change was generated by normalizing the transcript levels to those of healthy (H9) DE cells. Data represent the mean±s.e.m. [three technical replicates (three different wells) from each of the five biological cell lines differentiated at the same time]. (E) DE cells derived from healthy controls and patients similarly express CXCR4, GATA4 and SOX17 at the protein level, detected by immunofluorescence staining. Negative controls were included in each staining. Scale bars: 50 μm. ns, not significant.

The first critical step in generating esophagus epithelia is the differentiation of PSCs into endodermal cells that give rise to the entire epithelial lining of the gastrointestinal tract, including the esophagus epithelium (Wells and Melton, 1999). We evaluated the efficiency of endoderm differentiation by reverse transcription quantitative PCR (RT-qPCR) to analyze the gene expression levels of the specific markers CXCR4, GATA4 and SOX17 (Fig. 2A-D). There was no significant difference in gene expression levels between healthy and patient-derived definitive endoderms. At the protein level, CXCR4 and GATA4 were observed in the cytoplasm, whereas SOX17 was observed in cell nuclei, confirming definitive endodermal (DE) commitment in both groups (Fig. 2E). Furthermore, we also verified that no ectodermal cells were present through the absence of OTX2 (Table S5). Generated DE cells in both groups showed high Ct values by qPCR (39-40). We did not have a human positive control cell line expressing OTX2 such as the brain tissue to confirm the ectodermal commitment in both groups, in order to calculate DDCt values. Therefore, we relied on the high CT values to conclude that OTX2 was absent.

### The critical dorsal esophageal marker is downregulated in EA/TEF patient-derived anterior foregut cells

**Fig. 3.**
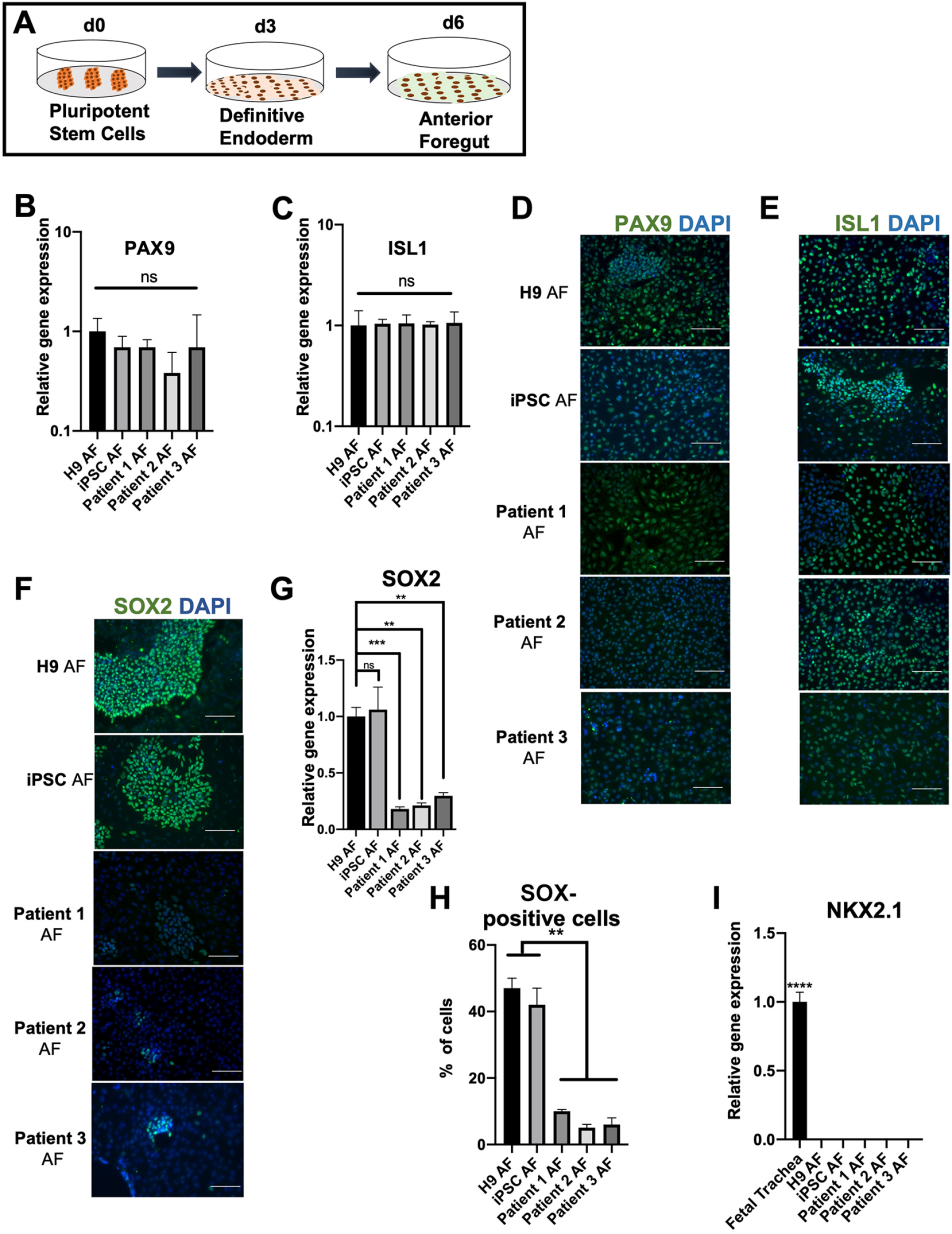
**SOX2 expression is significantly downregulated in patient-derived anterior foregut cells.** (A) Schematic representation of differentiation from the definitive endoderm into the anterior foregut (AF). (B,C) *PAX9* and *ISL1* are expressed similarly by RT-qPCR in both healthy and patient-derived anterior foregut cells. The fold change was generated by normalizing the transcript levels to those of healthy (H9) DE cells. (D,E) Healthy and patient-derived anterior foregut cells similarly expressed PAX9 and ISL1 nuclear staining, detected by immunofluorescence. (F) Low expression of SOX2 at the protein level was seen by immunofluorescence in all three patient-derived cells. Note that patients 2 and 3 had fewer cells with bright fluorescence and patient 1 had more cells with lower fluorescence. (G) *SOX2* was downregulated in all three patient-derived anterior foregut cells. Transcript levels were compared to those of H9 AF. (H) Cell counting of SOX2-positive cells using ImageJ software revealed that less than 10% of the patient AF cells were positive for SOX2. (I) Lack of expression of *NKX2.1* at the AF stage in both groups. The fold change was generated by normalizing the transcript levels to those of healthy (H9) DE cells. Data represent the mean±s.e.m. [three technical replicates (three different wells) from each of the five biological cell lines differentiated at the same time]. ns, not significant (*P*>0.05); **P≤0.01, ***P≤0.001; ****P≤0.0001; by unpaired two-tailed Student's *t*-test. Scale bars: 50 μm.

Developmentally, the anterior side of the foregut tube separates dorsally into the esophagus and ventrally into the trachea. Therefore, to generate the dorsal side of the anterior foregut, we inhibited key signaling pathways shown to be critical for esophagus specification: the BMP, TGFβ and WNT pathways (Fig. 3A). Anterior foregut cells in both groups expressed PAX9, a foregut endodermal marker, at the gene and protein levels (Fig. 3B,D). The cells also expressed ISL1 (Fig. 3C,E), a recently identified critical marker that contributes to the specification of the anterior foregut to both the esophageal and tracheal epithelia (Kim et al., 2019). ISL1 regulates the expression of NKX2.1 and is required for esophageal–tracheal separation (Zhang et al., 2018; Kim et al., 2019). The expression profiles of ISL1 and PAX9 were similar in both groups (Fig. 3D,E).

However, SOX2, a critical transcription factor necessary for foregut morphogenesis and expressed on the dorsal side of the anterior foregut, was downregulated in the patient-derived cells (Fig. 3F,G). Following quantification, we observed that SOX2 expression was significantly lower in all three patient-derived cells (∼10%) compared to the healthy foregut cells (45%) (Fig. 3F,H). It is known that disruption of SOX2 expression leads to an abnormal separation of the anterior foregut into the esophagus and trachea (Teramoto et al., 2020).

At the anterior foregut stage during which the compartmentalization occurs, two critical transcription factors, namely, SOX2 and NKX2.1, have a reciprocal repressive function. NKX2.1 binds to silencer sequences near the *SOX2* gene and represses its transcription (Kuwahara et al., 2020; Kim et al., 2019; Trisno et al., 2018; Han et al., 2020). However, dysregulated SOX2 did not affect the expression of *NKX2.1*, which remained undetected in both groups (Fig. 3I). Furthermore, at this stage, we also confirmed the absence of other lineage markers, specifically, the mid-hindgut marker CDX2 (data not shown) and the posterior foregut marker *HNF4A* (Fig. S1A).

### Novel transcript isoforms and distinct molecular signatures in patient-derived anterior foregut cells using nanopore sequencing

**Fig. 4.**
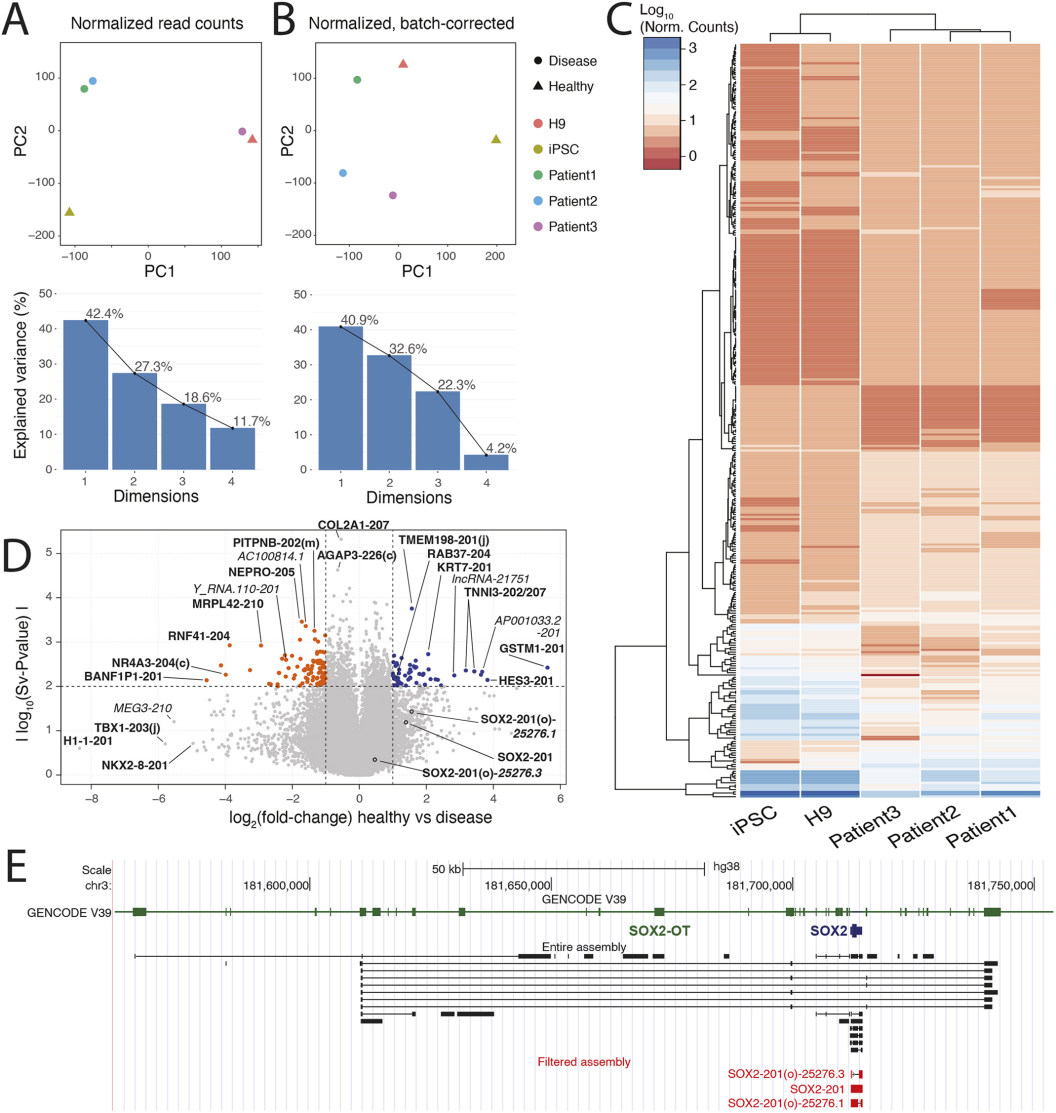
***De novo* assembly from long-read RNA sequencing of patient versus healthy cells.** (A,B) Principal component analysis of sequencing data before (A) and after (B) replicate- and sex-specific batch correction. (C) Heatmap of 173 transcript isoforms with batch-corrected *P*-value<0.01 and absolute fold change>2. (D) Volcano plot of differentially expressed transcripts (thresholds set at batch-corrected *P*-value=0.01 and log_2_(fold change)=±1. (E) UCSC Genome Browser view of the *SOX2* locus displaying collapsed reference transcriptome (top), all *de novo* assembled transcript isoforms from this study (middle) and filtered isoforms (bottom).

Low *SOX2* expression levels in patient-derived anterior foregut cells led us to investigate the other genes that might be involved in this stage of development. We thus applied RNA sequencing (Oxford Nanopore) on the anterior foregut cells derived from the two groups to quantify gene expression globally and characterize RNA transcript diversity across the surveyed samples. We report two new transcript isoforms of the *SOX2* gene, one of which presents an intron in its 3′UTR. This filtered *de novo* assembly was used as a reference for sample-specific abundance estimation. The latter revealed a potential batch effect associated with the dates on which the samples were prepared that, unfortunately, also coincided with the sex of the individual, representing 42% of the observed variation in the data [principal component 1 (PC1), Fig. 4A]. We performed batch correction using surrogate variable analysis (https://bioconductor.org/packages/release/bioc/html/sva.html) to mitigate this effect, which resulted in an effective separation of disease and healthy samples across the two remaining principal components (Fig. 4B). Sequencing validated our previous observations that *SOX2* has lower expression at the anterior foregut stage in the patient group. The differential transcript expression with DeSeq2 identified 173 transcripts that presented a >2-fold change in normalized expression, with a *P*-value below 0.01 (Tables S1 and S2). We could identify gene expression signatures unique to both conditions (Fig. 4C,D). Specifically, both *SOX2* transcripts that overlap the TaqMan probes used in RT-qPCR [SOX2-201 and SOX2-201(o)-25276.2, Fig. 4E] presented an average transcripts per million (TPM) value of 618 in healthy samples versus 253 in affected samples. GSTM1-201, a transcript isoform of *GSTM1*, was among the top differentially expressed isoforms in patient-derived cells [log_2_(fold change)=5.61]. Its levels were significantly lower in all three patients compared to healthy samples (Fig. 4D). Previous work has shown that *GSTM1* is associated with EA/TEF (Filonzi et al., 2010). Additionally, the *RAB37* variant RAB37-204 was also differentially expressed [log_2_(fold change)=1.08], which encodes an endosomal protein critical for vesicle trafficking regulation. Rab proteins have been previously linked to foregut malformations (Nasr et al., 2019; Nam et al., 2010; Edwards et al., 2021). Several non-coding RNAs were also present among the top differentially expressed transcripts, including *Y-RNA* and *MEG3* (Fig. 4D; Tables S1 and S2).

### NKX2.1, a tracheal marker, is expressed in EA/TEF patient-derived esophageal epithelia

**Fig. 5.**
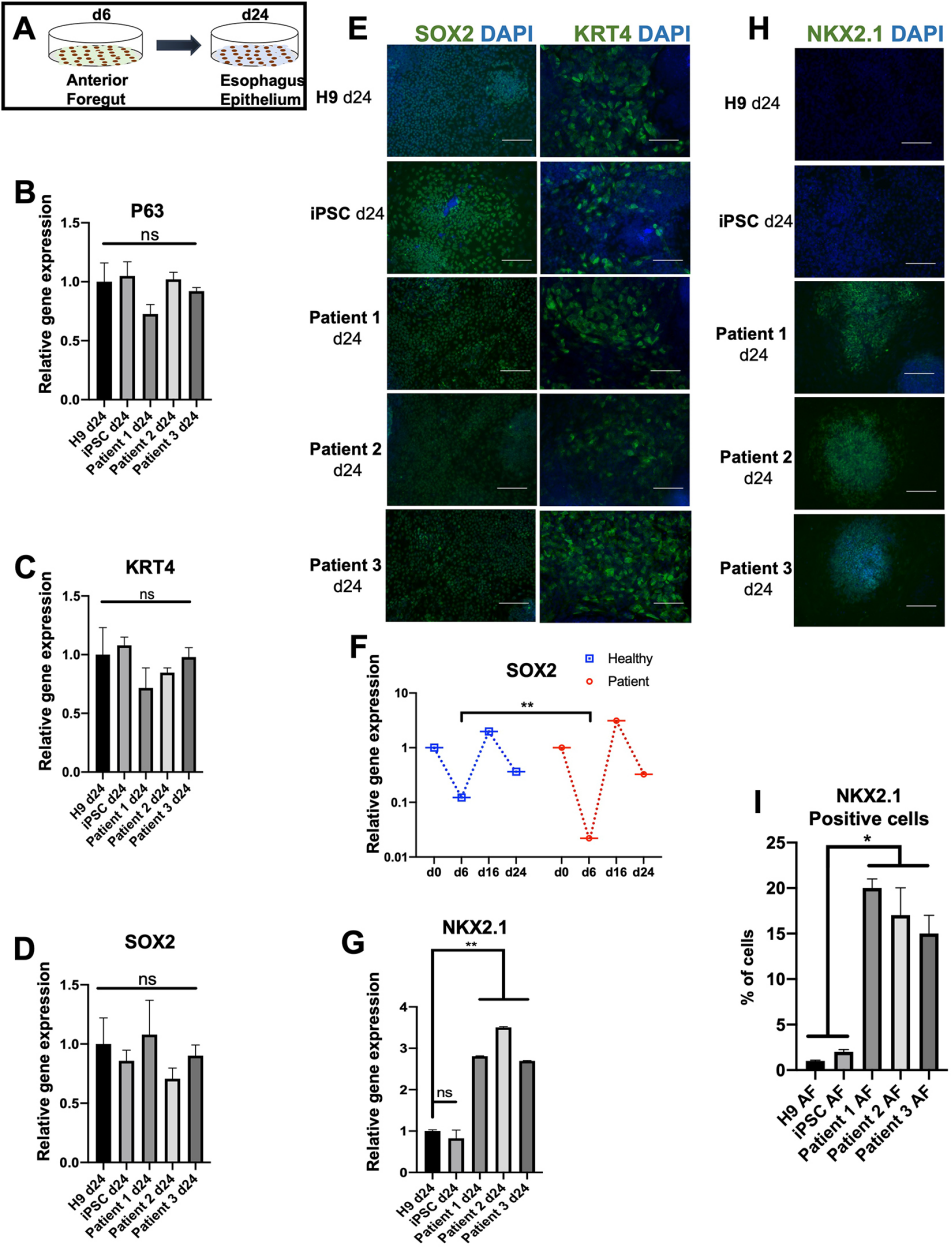
**Esophagus epithelial cells derived from EA/TEF patients express the tracheal marker NKX2.1.** (A) Illustration representing the differentiation of anterior foregut cells into the esophagus epithelium. (B-D) Transcript levels of *P63*, *KRT4* and *SOX2* showed similar expression in both healthy and patient groups. The expression was compared to that of H9 d24 esophagus epithelia. The fold change was generated by normalizing the transcript levels to those of healthy (H9) DE cells. (E) Immunofluorescence staining of d24 esophagus epithelia confirming the expression of SOX2 and KRT4 in healthy and patient-derived esophagus epithelia. (F) Relative expression of *SOX2* throughout differentiation. The fold change was generated by normalizing the transcript levels to those of healthy (H9) DE cells. (G) Abnormal expression of *NKX2.1* in patient-derived esophagus epithelia. The transcript levels were significantly higher in all three patient-derived cell lines. The transcript levels were compared to those of H9 esophagus epithelia. (H) At the protein level, NKX2.1 was also expressed in patient-derived esophagus epithelial cells, confirming our findings at the RNA level. (I) Cell counting of NKX2.1-positive cells by the ImageJ. Data represent the mean±s.e.m. [three technical replicates (three different wells) from each of the five biological cell lines differentiated at the same time]. ns, not significant (*P*>0.05), *P≤0.05; **P≤0.01; by unpaired two-tailed Student's *t*-test. Scale bars: 50 μm.

We further differentiated the anterior foregut cells into esophagus epithelia by inhibiting the BMP and TGFβ pathways (Que et al., 2006; Guyot and Maguer-Satta, 2016) (Fig. 5A). Even with low SOX2 expression in patient-derived anterior foregut cells, we observed that these cells were committed to an esophageal fate. Specifically, we observed that esophageal epithelial cells derived from both groups expressed the esophageal marker *P63* (or *TP63*), normally expressed in the basal proliferative layer of the developing esophagus (Fig. 5B). Cells from both groups also expressed keratin-4 (KRT4), an esophageal squamous epithelial marker (Fig. 5C,E). Interestingly, SOX2, a marker also expressed by the basal proliferative esophageal epithelium, was observed at similar levels in both groups (Fig. 5D,E).

The expression of *SOX2* during esophageal differentiation of EA/TEF patient iPSCs differed greatly from the healthy group. At the anterior foregut stage, we observed a temporal downregulation of SOX2 expression in the two groups, but it was significantly more pronounced in patient-derived cells (Fig. 5F). Interestingly, *SOX2* expression returned to similar levels to those of the healthy group at the esophageal epithelial stage (Fig. 5F). However, at this stage, although *SOX2* expression returned to normal levels, we observed a significantly higher expression of *NKX2.1* in patient-derived esophageal epithelial cells at the gene and protein levels (Fig. 5G,H). About 17% of the cells were positive for NKX2.1 in patient-derived esophageal epithelia (Fig. 5I). A recent study (Kim et al., 2019) identified ISL1 to be a regulator of NKX2.1 during foregut separation. However, we did not observe any significant difference in the expression of *ISL1* in both healthy and patient-derived cells at both the anterior foregut and esophageal epithelial stages (Fig. S1B).

### Mature esophageal epithelial organoids express the key markers involucrin, KRT4, KRT13 and P63

**Fig. 6.**
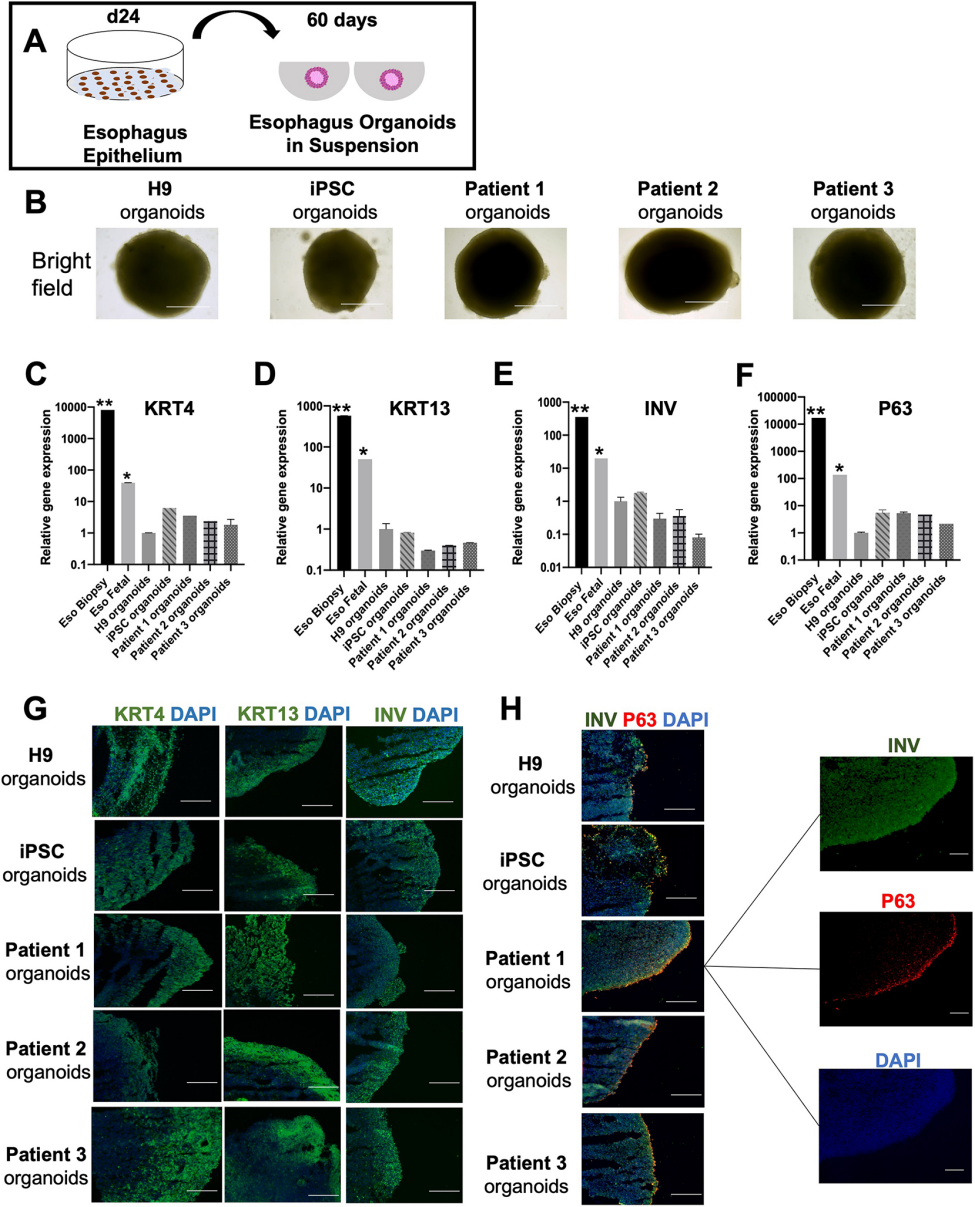
**Healthy and EA/TEF patient PSCs can generate mature esophagus organoids.** (A) Illustration of differentiation from esophagus epithelium progenitors into esophagus organoids. (B) Bright-field images of healthy and patient-derived esophagus organoids reveal similar morphology between the two groups. Scale bars: 250 μm. (C-F) Transcript levels of mature esophagus markers such as *KRT4*, *KRT13*, *INV* and *P63* reveal similar expression between healthy and patient-derived esophagus organoids. The expression was compared to H9-derived esophagus organoids. Two reference samples were included in the graphs, the esophagus (Eso) biopsy and fetal esophageal tissue. Data represent the mean±s.e.m. [three technical replicates (three different wells) from each of the five biological cell lines differentiated at the same time]. *P≤0.05; **P≤0.01; by unpaired two-tailed Student's *t*-test. (G) Immunofluorescence staining for KRT4, KRT13 and INV showing a positive expression in all generated esophagus organoids. (H) Dual immunofluorescence staining for INV and p63 showing a basal proliferative layer positive for p63 and a suprabasal layer positive for INV, a specific marker for stratified esophagus epithelium. Scale bars: 50 μm.

For further maturation and to allow for cellular organization of the esophageal epithelium into a stratified squamous epithelium, three-dimensional organoids were generated and further matured. Cells were detached from their two-dimensional culture conditions and matured in suspension (Fig. 6A), and within 48 h, the cells clustered together to form spheroids (Fig. 6B). After 2 months of culture, we observed no morphological and proliferative differences between the healthy and patient-derived organoids (Fig. 6B; Fig. S2). We observed high gene and protein expression of suprabasal markers such as KRT4, keratin-13 (KRT13), and involucrin (INV, encoded by *IVL*) in both healthy and patient-derived organoids at levels closer to those in the fetal esophagus than in adult esophagus biopsy (Fig. 6C-F). We also observed high levels of P63, a basal proliferative marker (Fig. 6G,H). We expected the expression of these markers to be normal at this stage because the upper and lower end of the esophagus in EA/TEF patients is not affected morphologically.

### Abnormal NKX2.1 expression is retained in EA/TEF patient-derived organoids

**Fig. 7.**
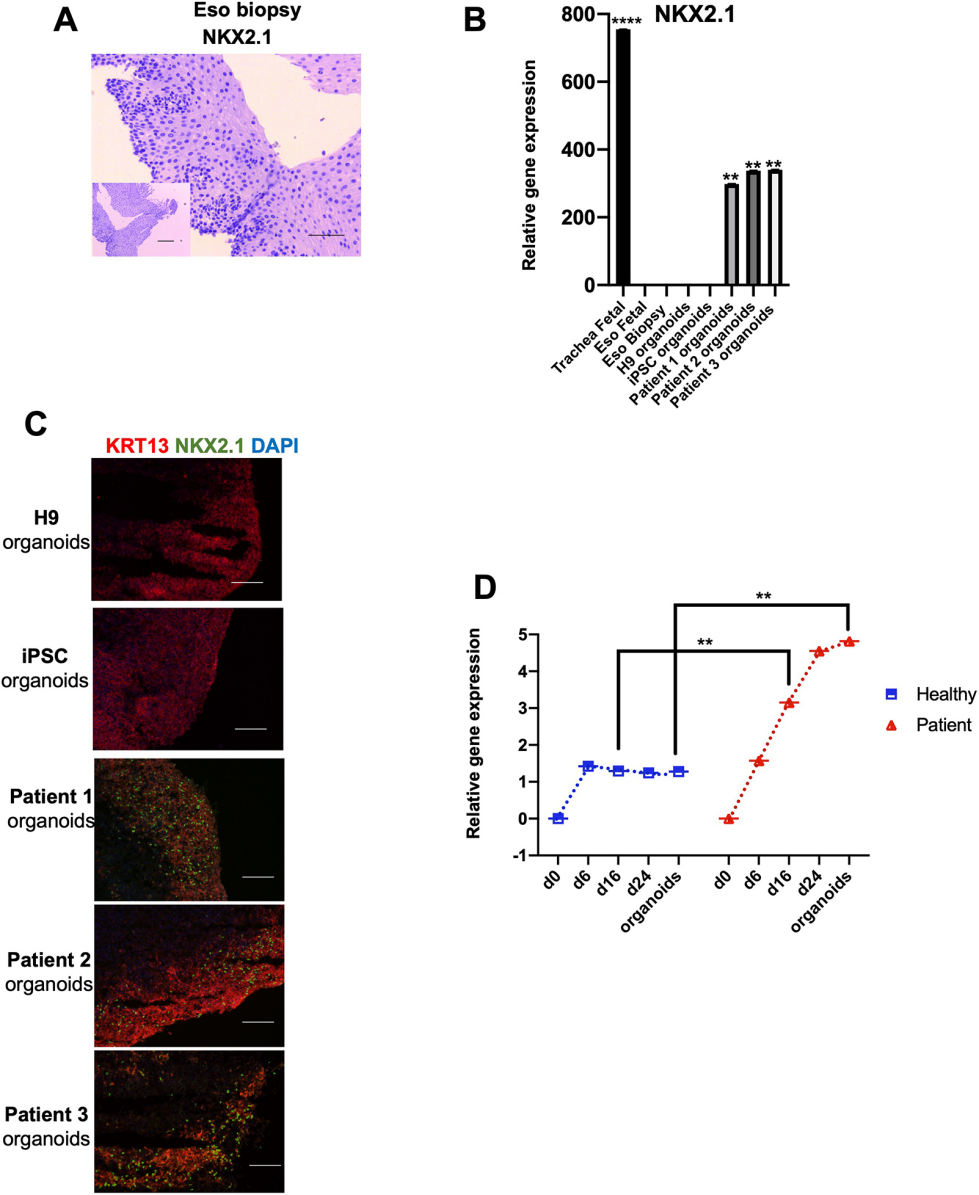
**Retained abnormal expression of NKX2.1 in patient-derived esophagus organoids reveals a similarity to fistulas connecting the esophagus to the trachea.** (A) Immunohistochemistry of esophagus biopsy against NKX2.1. Scale bar: 50 μm; 250 μm (inset). (B) Transcript levels of NKX2.1 were significantly higher in all three patient-derived esophagus organoids. The expression of NKX2.1 was absent in healthy control-derived organoids and esophagus biopsies. The transcript levels were compared to those of H9 derived organoids. Analysis of fetal trachea was included as a reference. (C) Dual immunofluorescence staining confirmed positive KRT13 staining in both groups; however, NKX2.1 was expressed in all three patient-derived esophagus organoids and was absent in healthy organoids. Scale bars: 50 μm. (D) Relative expression of *NKX2.1* throughout differentiation. The fold change was generated by normalizing the transcript levels to those of healthy (H9) DE cells. Data represent the mean±s.e.m. [three technical replicates (three different wells) from each of the five biological cell lines differentiated at the same time]. **P≤0.01; ****P≤0.0001; by unpaired two-tailed Student's *t*-test.

NKX2.1 is not normally expressed in human esophagus biopsies (Fig. 7A). However, our patient-derived esophageal organoids showed a positive expression of NKX2.1 at levels close to those in the fetal trachea. As expected, no expression was observed in healthy esophageal organoids, similar to the fetal esophagus and esophagus epithelial biopsies (Fig. 7B). NKX2.1 expression was interspersed in the KRT13-expressing suprabasal layers of the patient-derived esophageal organoids (Fig. 7C). A similar observation was made in TEF tissue from EA patients, which showed abnormal expression of NKX2.1 (Brosens et al., 2014). NKX2.1 dysregulation was detected as early as day 16 in esophagus progenitor cells (Fig. 7D). This abnormal expression was retained in mature organoids after 60** **days in culture (Fig. 7D).

### Differentiation propensity of EA/TEF iPSCs into different organ lineages is similar to that of healthy iPSCs

To verify whether the dysregulation of SOX2 at the anterior foregut stage and the abnormal expression of NKX2.1 at the mature esophageal epithelial stage in patient-derived iPSCs is specific to the esophageal fate, we investigated whether EA/TEF patient-derived iPSCs could be differentiated into other organ lineages such as tracheal, liver and muscle progenitor cells. By using a published protocol (Huang et al., 2014), we differentiated PSCs into ventral anterior foregut cells, thereby favoring a tracheal fate, which was confirmed by the expression of NKX2.1 at similar levels in both healthy and patient-derived groups (Fig. S3A,B). *SOX2* expression levels were also similar in the healthy and patient-derived tracheal epithelial cells (Fig. S3C).

We then differentiated patient-derived iPSCs into the posterior foregut and directed it towards the hepatic stage. All three EA/TEF patient-derived iPSCs generated hepatoblast cells. Patient-derived hepatoblasts expressed α-fetoprotein (AFP) (Fig. S4).

Finally, we directed the differentiation of the patient-derived iPSCs toward the mesodermal cell stage to generate skeletal muscle progenitor cells using a previously published protocol (Shelton et al., 2016). Myogenic progenitor cells derived from both healthy and patient-derived iPSCs expressed similar levels of PAX3 and PAX7, which are required for myogenic specification (Fig. S5). These results therefore suggest that any abnormal expression of key factors is intrinsic to the esophagus and not any other organ, as is observed in these type C EA/TEF patient-derived cells.

## DISCUSSION

We report here the first *in vitro*-generated, matrix- and xenogeneic-free, three-dimensional, mature stratified squamous esophageal epithelial organoids from EA/TEF patient-derived iPSCs. We observed a significant downregulation of *SOX2* mRNA and protein expression in patient-derived anterior foregut cells. Studies have shown that *SOX2* downregulation is linked to abnormal foregut separation, resulting in EA/TEF (Teramoto et al., 2020; Trisno et al., 2018; Ioannides et al., 2010; Que et al., 2007; Domyan et al., 2011). We also observed an abnormal expression of NKX2.1 in patient-derived cells at the esophageal epithelial stage until the organoid cultures. We also observed a distinct transcript expression profile in all three patient-derived anterior foregut cells, the most critical developmental stage during which patterning and subsequent separation into the esophagus and trachea occur. This dysregulation in gene and protein expression was specific to the dorsal side of the anterior foregut and, therefore, was specific of the esophageal fate. In fact, directed differentiation of EA/TEF iPSCs into posterior foregut-derived cells (hepatoblasts) and mesodermal cells (myoblasts) revealed similar gene and protein expression profiles to those of the healthy group.

The downregulation of SOX2 specifically, however, was temporary and SOX2 expression levels become similar in both groups when cells were further differentiated into the mature esophageal epithelium. The exact mechanisms regulating SOX2 expression in our patient-derived cells remain unclear. Although NKX2.1 and SOX2 are hypothesized to be co-repressive master regulators of foregut separation, NKX2.1 mRNA and protein levels remained unaffected at the anterior foregut stage. Interestingly, following nanopore sequencing, we observed an unannotated long non-coding RNA (*lncRNA-21751*) lying upstream of the *SOX2* promoter that was significantly downregulated in all three patient-derived anterior foregut cells. The exact role of *lncRNA-21751* in regulating SOX2 expression at the anterior foregut stage remains unknown. We also speculated on the potential role of the long non-coding RNA *SOX2OT* at this critical stage. *SOX2OT* harbors the intronic region of the *SOX2* gene. It plays a positive role in regulating SOX2 expression in a mechanism that remains largely unknown (Shahryari et al., 2014). Thus, we performed RT-qPCR analysis of *SOX2OT* on the anterior foregut cells and observed its expression to be downregulated in all three EA/TEF anterior foregut cells (Fig. S6). This downregulation of *SOX2OT* could be one of the regulatory mechanisms involved in the expression of SOX2 in the anterior foregut cells.

NKX2.1 is normally absent in the esophagus epithelium. However, in the patient-derived esophagus epithelia and organoids, NKX2.1 mRNA and protein levels were significantly high. ISL1, a recently identified transcription factor that regulates the expression of NKX2.1, was found at similar levels in both groups (Fig. S1B). Although information on the regulation of SOX2 expression is available, the mechanisms behind the upstream regulation of SOX2 at the earliest stages of anterior foregut development are unknown. At the anterior foregut stage, SOX2 and NKX2.1 have a reciprocal repressive function. NKX2.1 binds to silencer sequences near the *SOX2* gene and represses its transcription. In our experimental system, dysregulated SOX2 did not affect the expression of NKX2.1, which remained undetected in both groups, suggesting that NKX2.1 is not responsible for SOX2 downregulation at that stage. One could hypothesize that SOX2 expression is epigenetically regulated and/or lncRNAs (*lncRNA-21751*, *SOX2OT*) observed by RNA sequencing play a role in regulating SOX2 expression at the anterior foregut stage. It is also unknown whether lower SOX2 expression in the anterior foregut leads to abnormal esophageal development and abnormal maintenance of esophageal identity. A recent study suggests that mis-expression of Sox2 in gut precursors alters organ identity (Smith et al., 2022). The authors show that disruption of SOX2 expression is fully sufficient to alter cell fate decisions either by leading to a loss of identity or by completely changing cell fate. They further show that changes in key lineage-specific transcription factor binding events are sufficient to alter chromatin accessibility patterns and drive subsequent changes in lineage fate decisions.

Despite relatively shallow cDNA sequencing and the presence of a batch effect overlapping two experimental variables (biological sex and sample preparation date), we identified around 173 RNA transcript isoforms that were significantly differentially expressed between the healthy and patient groups. *GSTM1* was one of the most differentially downregulated genes, and the GSTM1 protein belongs to a family of enzymes that has distinct functions in the detoxification of electrophilic compounds including carcinogens, therapeutic drugs, environmental toxins, and products of oxidative stress (Cho et al., 2001). GSTM1 has a non-catalytic regulatory role in the apoptotic ASK1-mitogen-activated protein kinase (MAPK) signaling cascade (Cho et al., 2001). Under non-stimulated conditions, GSTM1 inhibits apoptotic cell death (Cho et al., 2001). There has been an increasing trend of linking xenobiotics to genes involved in detoxification in early embryonic development and specifically to EA/TEF. It is suspected that an altered detoxification process triggers an alteration of proliferation or apoptotic cellular behavior that may directly affect the separation process of the foregut into the esophagus and trachea (Filonzi et al., 2010). Another interesting gene which was differentially expressed was from the Rab family of small GTPases, which are key regulators of intracellular membrane trafficking. In a recent study, Rab11 was shown to have a direct link to epithelial remodeling and extracellular matrix degradation during the foregut separation (Nasr et al., 2019). The work shown in *Xenopus* and mouse demonstrates how the disruption of Rab11-mediated epithelial remodeling results in tracheoesophageal clefts (Nasr et al., 2019), providing a potential mechanistic framework for foregut separation in humans. In our patient-derived anterior foregut cells, however, we observed a significant downregulation of another Rab protein, RAB37. RAB37 is a critical regulator of vesicle trafficking and plays a potential role during human foregut compartmentalization, similar to what was observed in *Xenopus* and mice with Rab11. In a suggested mechanism, Rab37 mediates exocytosis of secreted frizzled related protein 1 (SFRP1), an antagonist of the WNT pathway, to suppress WNT signaling in lung cancer cells *in vitro* (Cho et al., 2018). The importance of the inhibition of WNT signaling in the anterior foregut to favor an esophageal fate (Woo et al., 2011) raises the potential role of Rab37 at this developmental stage. Furthermore, the identification of numerous new transcript isoforms, including known and previously unknown long non-coding RNAs, supports the observed regulatory complexity of esophagus and trachea development as well as EA/TEF etiology, and suggests that non-coding regulatory transcripts play a role in this process.

We cannot exclude a role of mesenchymal cells in the dysregulation of SOX2 and NKX2.1 in the present experimental setting. Although at minimal levels, we detected mRNA expression of brachyury (*TBXT*), which encodes a transcription factor that regulates mesoderm formation (Herrmann et al., 1990), and vimentin (*VIM*), encoding an intermediate filament expressed in mesenchymal cells, in our cultures during directed esophagus differentiation (Fig. S7A). Additionally, after 2 months of culture, we observed vimentin protein expression in our mature esophageal organoids derived from both healthy and patient cells (Fig. S7B). The influence of the mesenchyme on foregut epithelium division has been previously demonstrated (Han et al., 2020). Dysregulation of SOX2 has been linked to mesenchymal development with respiratory characteristics (Teramoto et al., 2020).

In conclusion, the experimental approach of using EA/TEF patient-derived iPSCs allowed us to mimic the initial developmental stages of the human esophagus to understand the origins of this malformation. We can conclude that the intrinsic defect observed in these cells are limited only to the esophagus. Our work is limited to isolated type C EA/TEF and thus we cannot relate our results to other types of EA or syndromic EA with associated malformations. More studies are required to understand the failure of key mechanisms and pathways involved during the critical stage of anterior foregut specification. This work therefore highlights the importance of using patient-derived iPSCs to model congenital diseases to yield new insights on organ development during embryogenesis.

## MATERIALS AND METHODS

### Blood collection for reprogramming

Blood was collected from three pediatric patients after obtaining consent from their parents to reprogram the blood cells to PSCs for research purposes. This study was approved by the Institutional Review Board of CHU-Sainte Justine Research Center [protocol #2018-1670 (for iPSCs); #2019-2102 (for whole exome sequencing)].

### Experimental design

Using the Institutional iPSC Core Facility, we reprogrammed peripheral blood mononuclear cells from three different EA/TEF type C patients. Control and patient cells were not matched for sex and ethnicity.

All iPSCs used for differentiation were between passages 20 and 35. Healthy and patient-derived iPSCs were differentiated simultaneously into mature esophageal organoids for every directed esophageal differentiation. The identity of the samples was not blinded to the investigator. Hepatic and myoblast differentiation were performed in the laboratories of Massimiliano Paganelli (CHU Sainte-Justine Research Center) and N.A.D., respectively. iPSCs derived from healthy subjects for esophageal differentiation, hepatic differentiation and myoblast differentiation were different. The same clones of EA/TEF patient-derived iPSCs were used for all directed differentiations.

### Human embryonic stem cell and induced pluripotent stem cells

The human ESC line H9 was a kind gift from the laboratory of Gregor Andelfinger at CHU Sainte-Justine Research Center (Wünnemann et al., 2020). The healthy iPSC cell line (GHC4) and EA/TEF patient-derived iPSCs (EA1, EA2 and EA3) were generated and obtained from the iPSC Core Facility at CHU-Sainte Justine Research Center (see Raad et al., 2022 for details of patient-derived cell lines). Patient 1 was a 2-year-old male, patient 2 was a 6-year-old male and patient 3 was an 18-year-old female.

### Culture and expansion of ESCs and iPSCs

Both ESCs and iPSCs were cultured on feeder-free and non-xenogeneic conditions. Cells were plated on human vitronectin VTN XF (STEMCELL Technologies, 100-0763)-coated 100 mm cell culture dishes. Cells were maintained at 37°C with 5% CO_2_ with daily replacement of Essential 8 (E8) medium system (Thermo Fisher Scientific, A1517001). Cells were passaged as aggregates every 3-4 days with 0.5 mM EDTA diluted in PBS (Thermo Fisher Scientific, 15575020) until they reached 60-70% confluency.

### Differentiation protocol

#### Preparing cells for differentiation (days −1 and 0)

Two days prior to differentiation (D−1), cells were dissociated into single cells using Accutase (STEMELL Technologies, 07922) and transferred onto Biolaminin 521 LN (LN521; BioLamina, Sweden, LN521-02)-coated plates with E8 medium supplemented with 10 μM Rock inhibitor Y-27632 (Sigma-Aldrich, SCM075). The following day (D0, 12-16 h later), the medium was changed to the E8 medium only. If the survival rate of the cells was less than 50%, they were cultured for an additional 24 h before starting the differentiation. ESC and iPSCs were maintained at 37°C with 5% CO_2_ throughout the differentiation process.

#### Endoderm differentiation (day 1 to day 3)

We modified and adapted the previously published protocol for endoderm differentiation (Matsuno et al., 2016). Xeno-free medium (XFM–) was prepared using 500 ml of RPMI 1640 medium without L-glutamine (Thermo Fisher Scientific, 11875101), 10 ml B-27 supplement, minus insulin (Thermo Fisher Scientific, A1895601), 5 ml GlutaMax (Thermo Fisher Scientific, 35050061), 5 ml KnockOut serum replacement (Thermo Fisher Scientific, 10828010), 5 ml penicillin-streptomycin (10,000 U/ml) (Thermo Fisher Scientific, 15140148), 7.5 ml HEPES (1 M) buffer (Thermo Fisher Scientific, 15630130) and 5 ml of MEM non-essential amino acids (100×) (Thermo Fisher Scientific, 11140050). Day 1 of differentiated cells were first washed with XFM– to remove any residual E8 medium, then cultured in XFM– with 100 ng/ml activin A (R&D Systems, 338-AC-010/CF) and 3 μM CHIR99021 (STEMCELL Technologies, 72052). On days 2 and 3 of culture, cells were first washed with XFM– and the culture was continued with XFM– supplemented with 100 ng/ml activin A and 250 nM of LDN193189 (Stemgent, 04-0074). By day 3, a 70-80% confluent monolayer of endodermal cells could be observed under the microscope.

#### Anterior foregut differentiation (days 4 and 5)

Endodermal cells were first washed with XFM– and then cultured for 24 h in XFM– supplemented with 1 μM A8301 (Stemgent, 04-0014) and 250 nM of LDN193189. The following day, cells were washed in XFM– and cultured in XFM– supplemented with 1 μM A8301 and 1 μM IWP2 (Stemgent, 04-0034).

#### Esophagus differentiation (day 6 to day 24)

From day 6 to day 16, we switched to XFM+, which contained the same components as XFM– except that the B-27 supplement without insulin was replaced with a B-27 supplement with insulin (Thermo Fisher Scientific, 17504044). To induce esophageal fate, we modified and adapted a previously published protocol (Zhang et al., 2018; Trisno et al., 2018). Anterior foregut cells were cultured in XFM+ supplemented with 1 μM A8301 and 250 nM LDN193189 from day 6 until day 16, and the medium was changed daily. By day 16, we observed that cells had reached 100% confluency and the presence of dense cell clusters. After day 16, esophageal progenitor cells were cultured in XFM+ only until day 24.

#### Esophageal organoid formation (2 months)

Organoids were generated in suspension using Nunclon Sphera low attachment 96-well plates (Thermo Fisher Scientific, 174930) by modifying previously published esophageal studies (Giroux et al., 2017; DeWard et al., 2014). On day 24 of esophageal differentiation, cells were detached using TrypLE (Thermo Fisher Scientific, 12604013) and gently resuspended in XFM+. Viable cells were counted using Trypan Blue solution (Thermo Fisher Scientific, 15250061) and ∼50,000 cells were then aliquoted in each well of the 96-well plate containing XFM+ supplemented with 1 μM A8301, 250 nM LDN, 3 μM CHIR99021, 20 ng/ml FGF2/bFGF (PeproTech, AF100-18B) and 200 ng/ml EGF (Thermo Fisher Scientific, PHG0313).

### Other organ-lineage differentiation

#### Trachea differentiation (day 6 to day 16)

To induce tracheal fate, we again modified previously published protocols (Huang et al., 2014, 2015). Anterior foregut cells were cultured from day 6 to day 16 in XFM+ supplemented with 3 μM CHIR99021, 10 ng/ml human FGF10 (R&D Systems, 345-FG-025/CF), 10 ng/ml human FGF7 (R&D Systems, 251-KG-010/CF), 10 ng/ml BMP4 (PeproTech, 120-05) and 50 nM retinoic acid (Tocris, 0695), and the medium was changed daily. Cells reached 100% confluency and formed two-layered cell clusters.

#### Hepatoblast differentiation (day 0 to day 15)

iPSCs were dissociated by TrypLE (Thermo Fisher Scientific, 12604013) to single cells and seeded on human recombinant laminin 521 (BioLamina)-coated plates in Essential 8 Flex medium (Thermo Fisher Scientific, A1517001) at a density of 7×10^5^ cells/cm^2^. Differentiation was started (day 0) when the cells reached around 70% confluency by changing the medium to RPMI B-27 minus insulin (Life Technologies) supplemented with 1% knockout serum replacement (KOSR, Life Technologies, 10828010). For the first 2 days, the cells were exposed to 100 ng/ml activin A and 3 μM CHIR99021, and then to 100 ng/ml activin A alone for the following 3 days. Subsequently, RPMI B27 minus insulin medium was supplemented with 20 ng/ml BMP4, 5 ng/ml bFGF (PeproTech, AF100-18B), 4 μM IWP2 (Tocris, 3533) and 1 μM A83-01 (Tocris, 2939/10) for 5 days, and the medium was changed daily. At day 10, the medium was changed to RPMI B27 (Life Technologies, 17504044), supplemented with 2% KOSR, 20 ng/ml BMP4, 5 ng/ml bFGF, 20 ng/ml HGF (PeproTech, 100-39H) and 3 μM CHIR99021 for 5 days, and the medium was changed daily.

#### Myoblast differentiation (day 0 to day 20)

The generation of iPSC-derived myoblast was adapted from a protocol published by Shelton et al. (2016) with minor modifications. Briefly, different growth factors and inhibitors are sequentially used to drive iPSCs toward the mesodermal lineage and promote their myogenic cell fate commitment. One day prior to differentiation, human iPSCs (patient-derived and control) were dissociated with TrypLE (Thermo Fisher Scientific, 12604013) and 10^5^ cells/well were plated as small colonies (10-20 cells/colony) on vitronectin-coated 12-well plates using mTeSR1 medium (STEMCELL Technologies, cat. no. 85850) supplemented with 10 μM ROCK inhibitor (Y-27632, STEMCELL Technologies, cat. no 72302). The next day, the medium was changed to TeSR-E6 medium (STEMCELL Technologies, 05946) supplemented with 7 μM CHIR99021 (STEMCELL Technologies, 72052) and the cells were cultured for 3 days. After 3 days of CHIR99021 treatment, cells were gently washed with DPBS (STEMCELL technologies, 37350) and cultured only in TeSR-E6 medium without any CHIR99021, and the medium was changed every day until day 7. At this time point, a broad expression of the somite markers PAX3 and MEOX1 could be detected. From day 10 to day 20 of differentiation, 5 ng/ml FGF2 (Wisent, cat. no. 3718-FB-010) was added to the TeSR-E6 medium to promote myogenic cell proliferation. At day 20, a significant proportion of cells expressed the muscle stem cell marker PAX7.

### RT-qPCR

At each developmental stage (definitive endoderm, anterior foregut, esophageal progenitor, mature esophageal epithelium/organoids and other organ lineages), cells were detached using Accutase and RNA was extracted using the ReliaPrep RNA Cell Miniprep System (Promega, Z6011). RNA was reverse transcribed using the Omniscipt RT kit (QIAGEN, 205113) and cDNA obtained was used for real-time quantitative PCR using a LightCycler instrument (Roche Life Science, Germany). cDNA was quantified using TaqMan Gene expression assays and the TaqMan primers to target genes (purchased from Thermo Fisher Scientific) are listed in Table S3. The transcript level of each gene was normalized to the housekeeping gene *GAPDH* using the 2^–ΔΔCT^ method. Relative gene expression was calculated and reported as fold change compared to the indicated samples using *GAPDH*-normalized transcript levels. The results include a mean of at least three technical replicates for each biological sample.

### Immunofluorescence and microscopy

For immunofluorescence staining, cells at each developmental stage (definitive endoderm, anterior foregut, esophageal progenitor, mature esophageal epithelium/organoids and other organ lineages) were fixed with 4% paraformaldehyde (Thermo Fisher Scientific, AAJ19943K2) for 20 min at room temperature, then washed three times with DPBS. Cells were then permeabilized with 0.4% Triton X-100 (Sigma-Aldrich, 9002-93-1) in DPBS for 25 min at room temperature, followed by washing with DPBS. Cells were then incubated with 3% blocking serum in DPBS for 1 h at room temperature. The primary antibody was added to the antibody dilution buffer (PBS with 1% bovine serum albumin, 0.3% Triton X-100 and 0.3% serum) and the samples were incubated overnight at 4°C. The following day, the cells were washed three times with DPBS. The secondary antibody was diluted in the same antibody dilution buffer, added to cells and incubated for 1 h at room temperature. Following washing with DPBS, cells were stained with DAPI for 15 min at room temperature. Cover slips were mounted on top of a drop (7-8 μl) of ProLong Diamond Antifade Mountant (Thermo Fisher Scientific, P36970). The primary and secondary antibodies used are listed in Table S4.

### Immunohistochemistry

NKX2-1 immunohistochemistry was performed on paraffin-embedded sections using an automated Ventana immunomarker (Benchmark, XT Ventana Medical System Inc., Tucson, AZ). This was done according to the company's protocol and by using the NKX2-1 monoclonal antibody (Table S4).

### RNA-sequencing assay

RNA was extracted as described in the ‘RT-qPCR’ section above. Library preparation for Nanopore Sequencing was done using two different protocols. A total of 50 ng RNA was reverse transcribed and amplified using the cDNA-PCR Sequencing kit (SQK-PCS109, Oxford Nanopore Technologies, UK) following the manufacturer's instructions, up until the PCR step (14 cycles, 500 s extension). The PCR reactions were then prepared for sequencing using the Genomic DNA by Ligation kit (SQK-LSK109, Oxford Nanopore Technologies). Concentrations were quantified for RNA after elution, for cDNA after the PCR step, and before loading the flow cells using a Qubit Fluorometer with a Qubit RNA assay kit (high sensitivity) for RNA and a Qubit dsDNA assay kit (broad range) for cDNA (Invitrogen). The final libraries [with the following cDNA concentrations: 26.2 ng/μl (patient 1), 29 ng/μl (patient 2), 23.8 ng/μl (patient 3), 28 ng/μl (iPSCs) and 13.4 ng/μl (H9)] were loaded onto MinION flow cells (R9.4.1, FLO-MIN006, Oxford Nanopore Technologies) and ran for 74 h on GridION and MinION Mk1C sequencers (Oxford Nanopore Technologies). When required, the sequencing runs were refueled with 250 μl of FB buffer (Flow Cell Priming Kit EXP FLP002).

### RNA-sequencing analysis

Raw FAST5 files were basecalled during the sequencing run using Guppy v4.0.11 (https://nanoporetech.com/) in high-accuracy mode. Fastq_pass and fastq_fail files for each sample were submitted to Pychopper v2.4.0 (https://github.com/nanoporetech/pychopper) to identify full-length reads, split ligation concatemers, rescue fused reads and reorient reads based on the stranded barcode adapters (Pychopper full length as well as rescued reads for all processed samples are available on the European Nucleotide Archive under the project accession ID PRJEB55419). Full-length and rescued reads were then aligned to the human reference genome (GRCh38.p13) (Frankish et al., 2019) using Minimap2 v2.18 (Li, 2018) with -a -x splice –MD –secondary=no options. Alignments were converted to BAM format and sorted with Samtools v1.12 (Li et al., 2009). The resulting BAM files were merged using ‘samtools merge’ before being used as input for *de novo* assembly with Stringtie2 v2.1.4 (Kovaka et al., 2019). The Gencode reference transcriptome v37 gtf format was used as input for the -G option in Stringtie2, and all transcripts were collapsed with the long reads -L parameter. GFFcompare v0.1.12.2 (Pertea and Pertea, 2020) was used to map the resulting GTF file to Gencode to evaluate the assembly and filter it. TPM, classcode and exon number filters were applied manually to select all isoforms that were TPM>0.2, ‘=’ or ‘c’ classcode (provided by GFFcompare) and all classcode of more than one exon. FASTA sequences corresponding to the filtered assembly annotation were retrieved using GFFread v0.12.7 (Pertea and Pertea, 2020) and the sample FASTQ sequences obtained after Pychopper were aligned again using minimap2 with -k 14 -a -N 100 option and the FASTA sequence from the filtered assembly as a reference. BAM files were obtained using Samtools and isoforms were quantified using Salmon v1.5.2 (Patro et al., 2017) in quant mode with the -l SF –noErrorModel –noLengthCorrection options as recommended for Nanopore long reads. Read counts from all samples were merged into the same matrix using the Salmon quantmerge option. Isoforms with more than three samples with null read counts were filtered out manually to avoid artefacts. Normalization and differential expression analysis was performed using DESeq2 (Bioconductor version 3.13) (Love et al., 2014) on R v4.1.0 (https://www.R-project.org/) to generate a normalized count matrix and statistics on differential expression. Batch effect correction was done using the SVA package (Leek et al., 2021). Isoforms with *P*<0.01 and a |log_2_(FoldChange)|>1 after batch correction were considered statistically significant in the differential expression analysis. Isoforms with a *P*<0.01 and |log_2_(FoldChange)|>0.5 were used to perform a Gene Ontology enrichment analysis with GOrilla (Eden et al., 2009).

### Quantification and statistical analysis

All data quantification is presented as the mean±s.e.m. using GraphPad Software Prism 6. Statistical significance was determined by Student's *t*-tests and Mann–Whitney test. For each analysis, at least three technical replicates of each biological cell lines were included. Details of the representative pictures shown are indicated in the legends. *P*-values of 0.05 or less were considered statistically significant.

## Supplementary Material

10.1242/dmm.049541_sup1 Supplementary information
