# Supplementary material for: iPSCs derived from esophageal atresia patients reveal SOX2 dysregulation at the anterior foregut stage
Source: Dis Model Mech. 2022 Nov 28;15(11):dmm049541. doi: 10.1242/dmm.049541 (PMC10655818; doi:10.1242/dmm.049541)
Supplement: Supplementary information [file dmm-15-049541-s1.pdf]

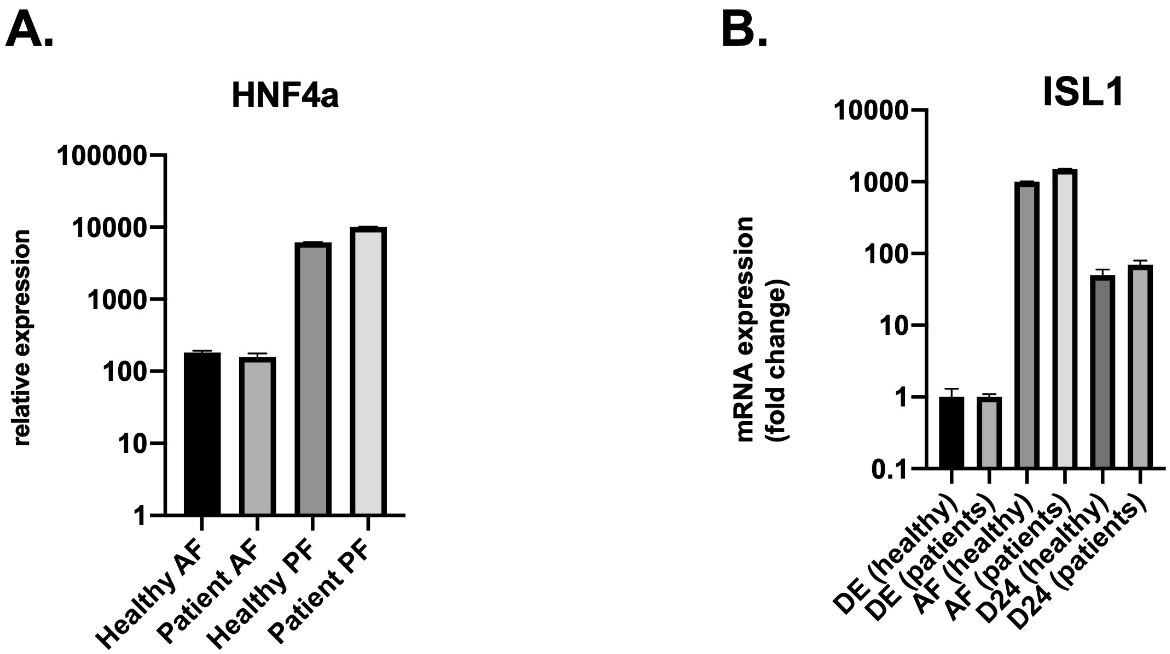

Fig. S1. mRNA expression levels of HNF4a, ISL1 in healthy and patient derived cells. A) Expression levels of HNF4a, a posterior foregut marker, by qPCR is absent in healthy and patient derived anterior foregut cells compared to posterior foregut cells. mRNA levels were represented by the fold change compared to healthy posterior foregut cells**. B)** Expression of ISL1 mRNA levels by qPCR at definitive endoderm, anterior foregut and esophagus epithelium at day 24 are similar between healthy and patient cells. Fold change relative expression was compared to healthy definitive endodermal cells. (**3 technical replicates from 1 well for each of the 5 biological cell lines. Posterior foregut and anterior foregut differentiations were conducted at different time points)**

Disease Models & Mechanisms • Supplementary information

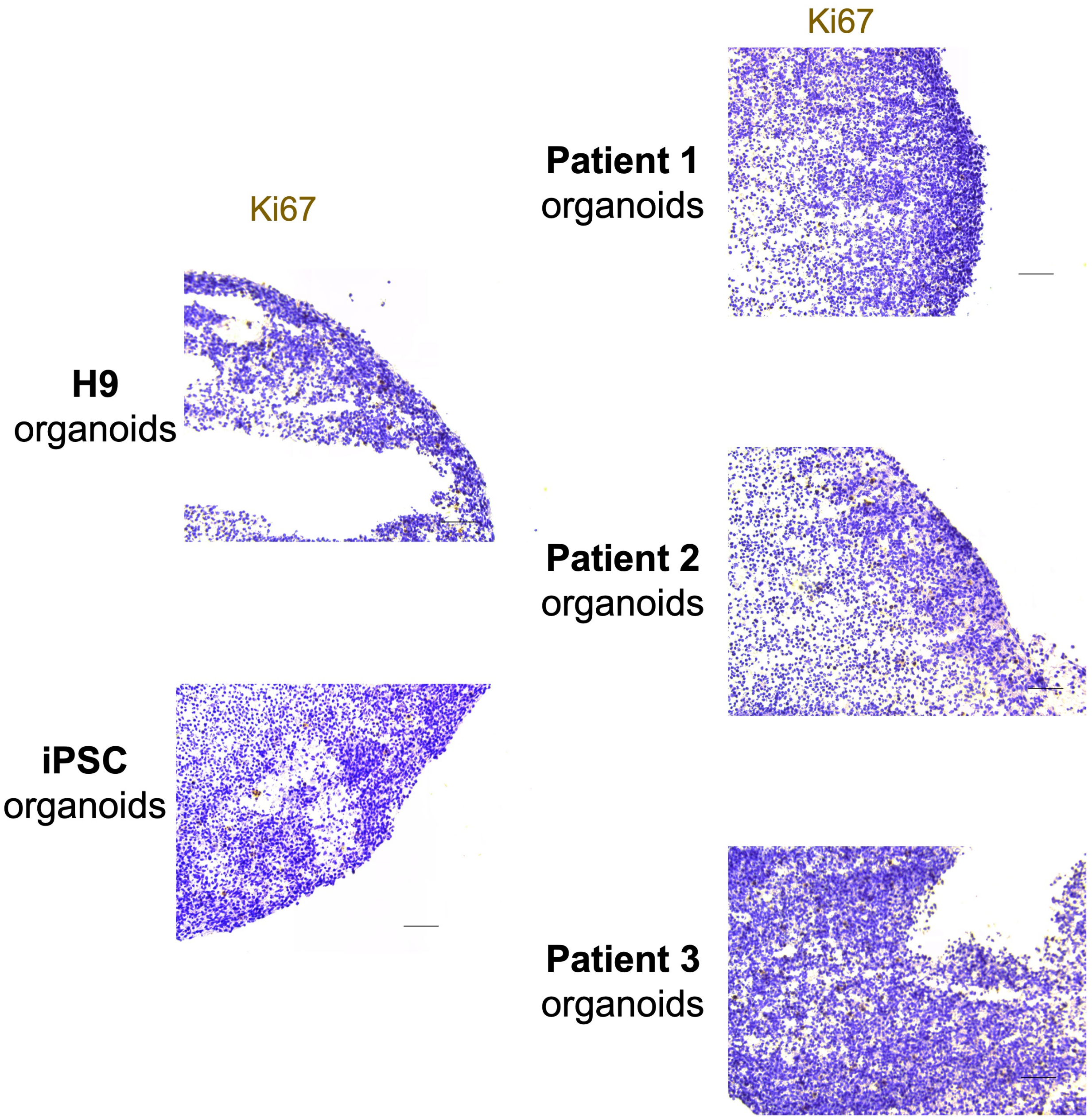

Fig. S2. Healthy and Patient-derived organoids can proliferate after 2 months of culture. Immunohistochemical staining for ki67, a proliferative marker, shows a similar proliferative capacity in both healthy and patient derived organoids after 2 months of culture. Scale bar 50um

Disease Models & Mechanisms • Supplementary information

A.

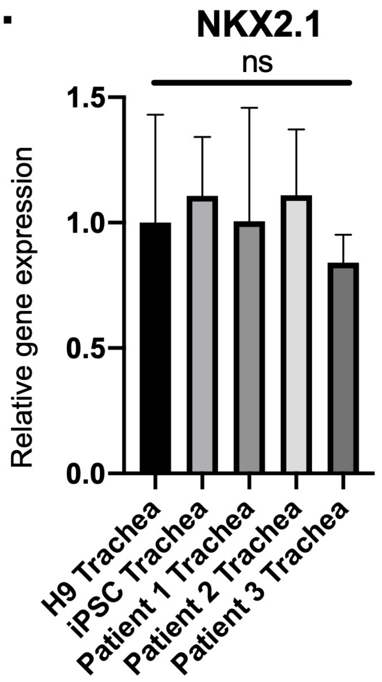

B.

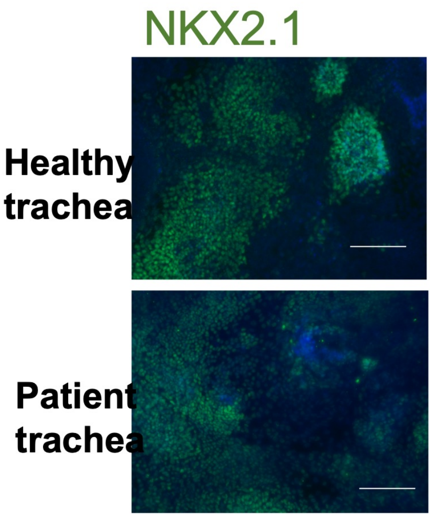

C.

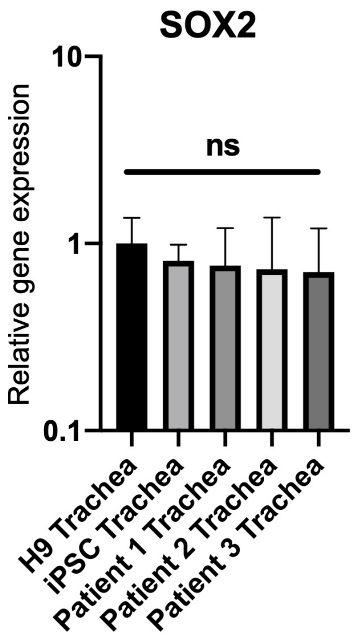

Fig. S3. Healthy and Patient-derived iPSCs can generate tracheal epithelium. A) Expression of NKX2.1 transcription factor in tracheal epithelial cells derived from healthy and patient iPSCs reveal similar expression levels. mRNA relative expression was compared to the healthy group. Data represent mean +- SEM (**3 technical replicates from 1 well for each of the 5 biological cell lines differentiated at the same time). B)** Immunofluorescence

staining of tracheal epithelium from healthy and patient cells reveal expression of NKX2.1 in both groups. Negative controls were included for each staining. Scale bar 50um. **C**) Expression of SOX2 transcription factor in tracheal epithelial cells derived from healthy and patient iPSCs reveal similar expression levels. mRNA relative expression was compared to the healthy group. Data represent mean +- SEM (**3 technical replicates from 1 well for each of the 5 biological cell lines differentiated at the same time**).

Disease Models & Mechanisms • Supplementary information

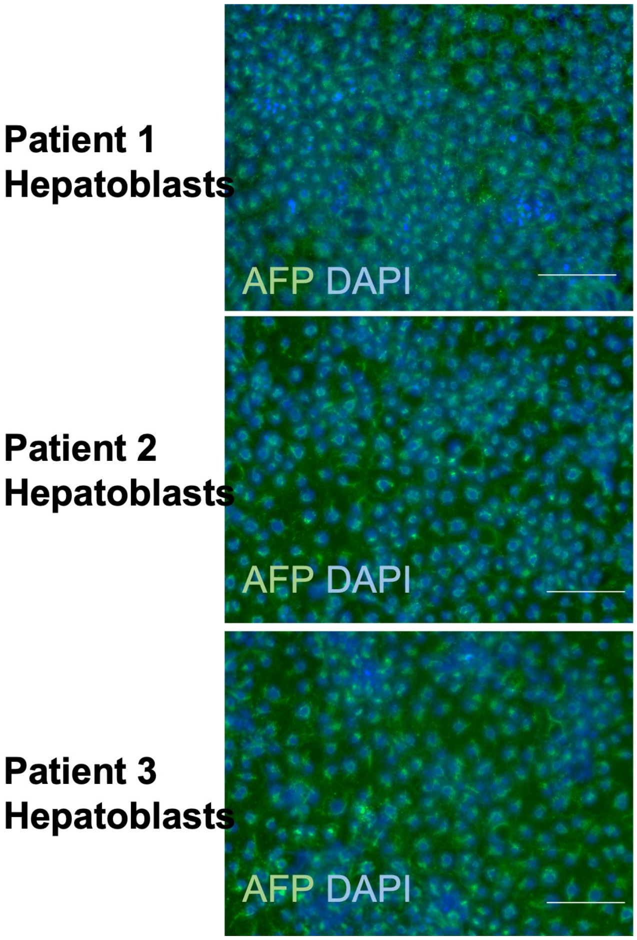

Fig. S4. Hepatic differentiation of healthy and patient derived iPSCs. Immunofluorescence staining of all 3-patient derived hepatoblasts reveal expression of AFP a specific hepatic marker. Scale bar 50um

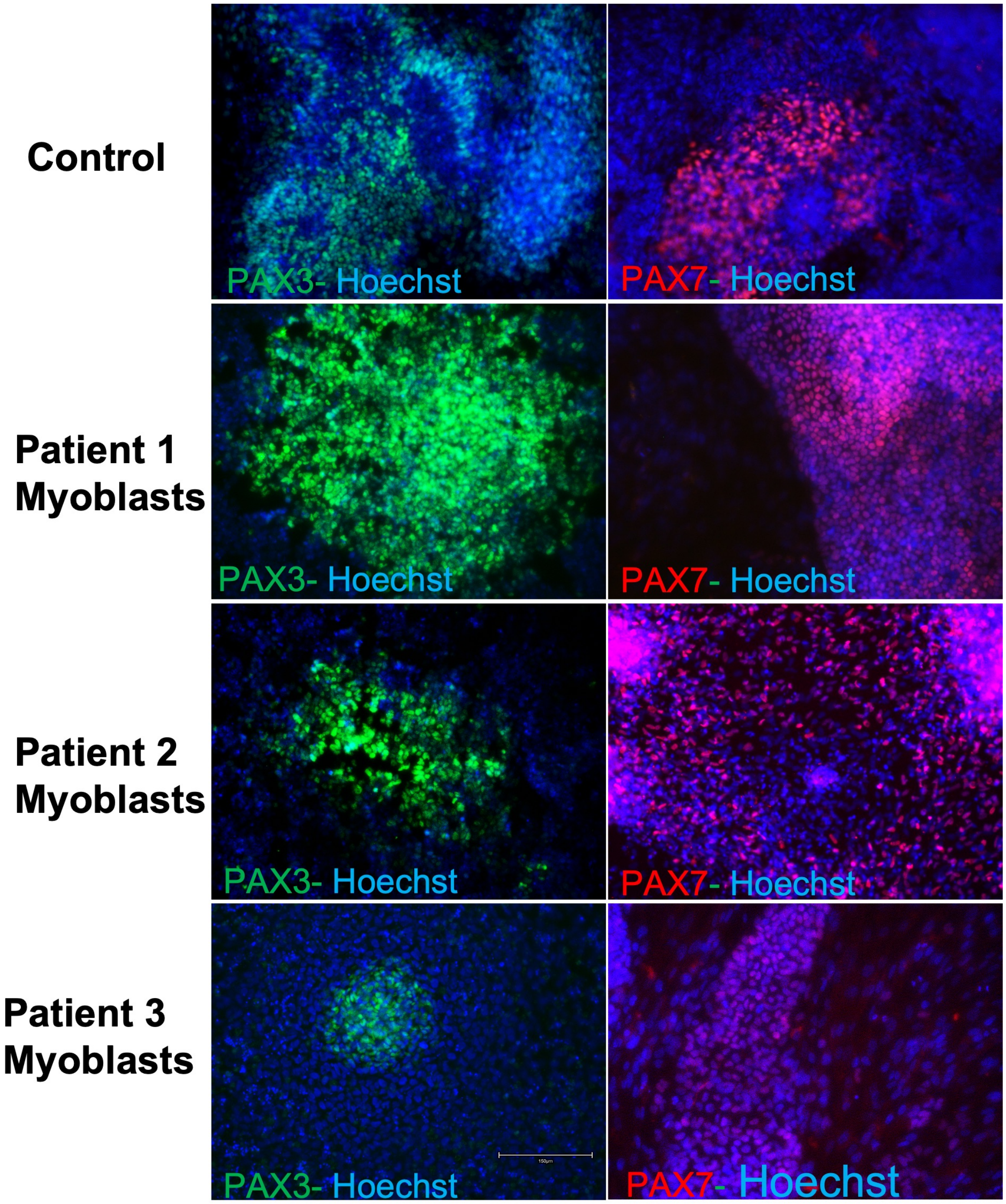

Fig. S5. Differentiation of iPSCs into myoblast cells.

Immunofluorescence staining of 3 patient-iPSC derived myoblasts reveal similar protein expression of PAX3 and PAX7 similarly to the control group. Scale bar 100um

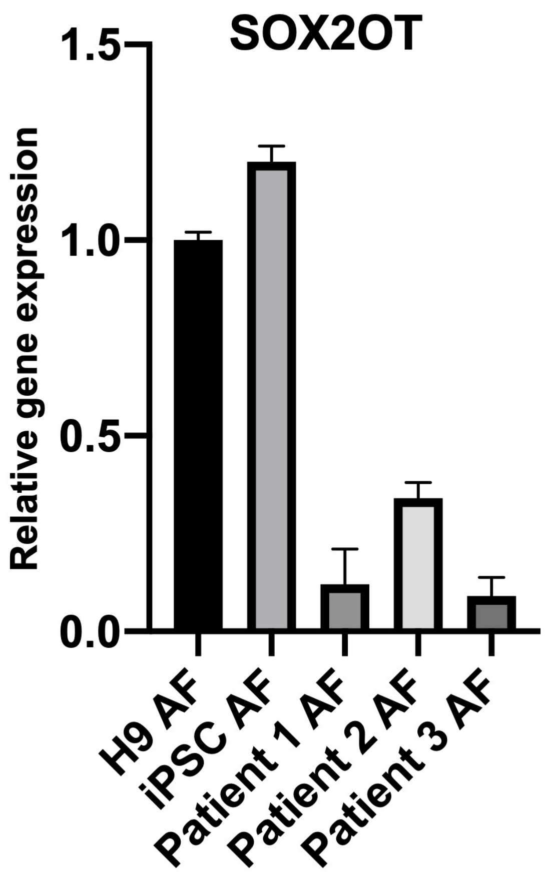

Fig. S6. Long non-coding RNA SOX2OT a potential regulator of SOX2 at the anterior foregut stage.

mRNA levels by qPCR reveal significant downregulation of SOX2OT in all 3 patient-derived anterior foregut cells. Relative expression was compared to H9 AF cells. (**3 technical replicates from 1 well for each of the 5 biological cell lines differentiated at the same time**).

Disease Models & Mechanisms • Supplementary information

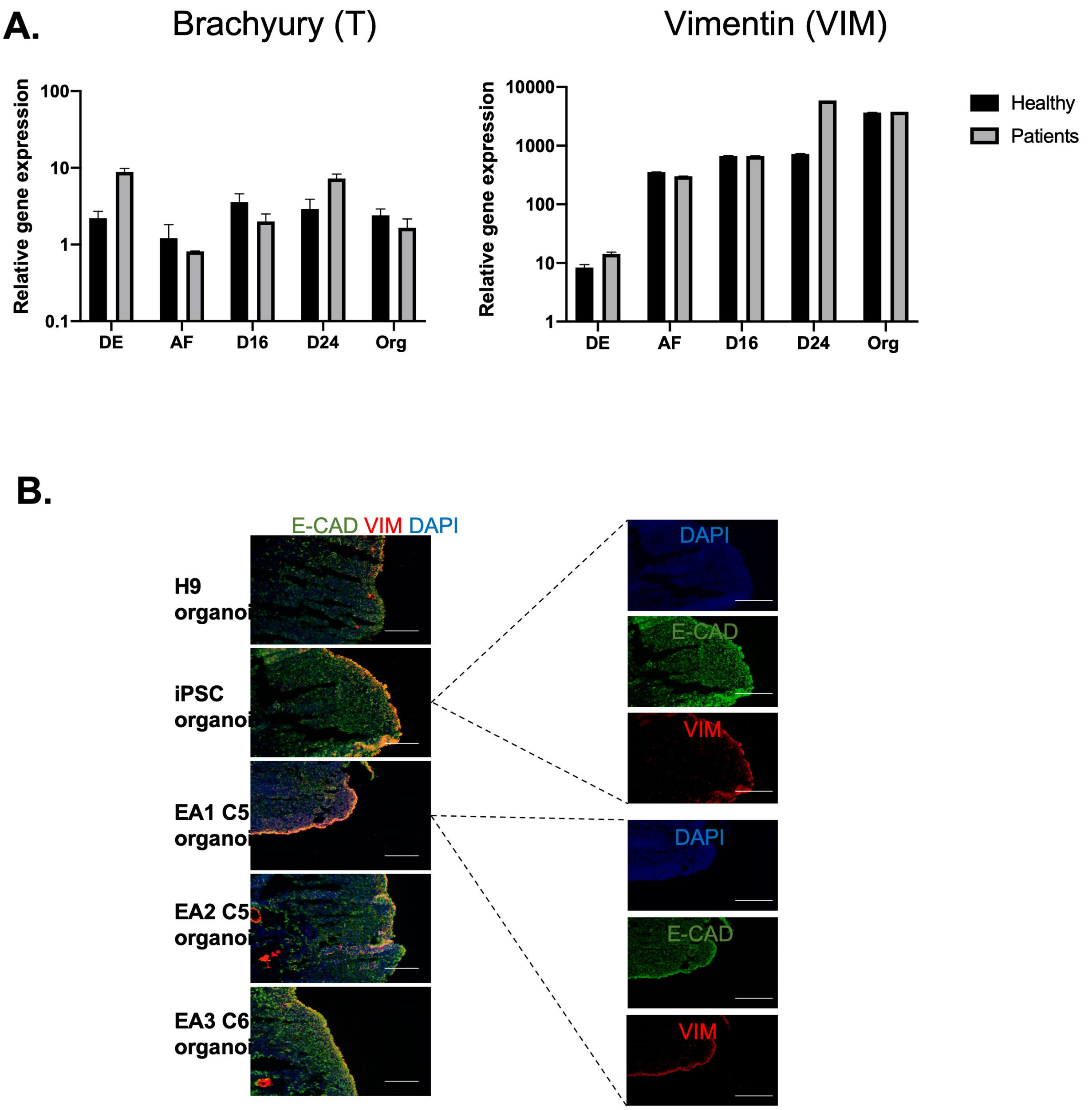

Fig. S7. 2 months mature esophagus organoids express mesenchymal markers brachyury and vimentin. A) Transcript levels of Brachyury (T) and Vimentin (Wim) throughout esophagus differentiation is similar in both healthy and patient derived cells. Transcript levels were compared to those of day 0 iPSCs (**3 technical replicates from 1 well for each of the 5 biological cell lines differentiated at the same time) B**) Healthy and patient derived esophagus organoids express by immunofluorescence vimentin VIM in the outermost layer of the esophagus whereas the rest of the esophagus is positive for E-cadherin, an epithelial marker. Esophagus organoids are organized with an inner core of epithelial cells and outer layer of mesenchymal cells. Scale bar 50um

Disease Models & Mechanisms • Supplementary information

Table S1. 40 top differentially expressed transcripts in patient-derived anterior foregut cells. Long read single molecule cDNA sequencing analysis revealed a list of 173 transcripts differentially expressed in patient derived anterior foregut cells. The 40 most differentially expressed are described here (40 transcripts with the highest fold change value and batch-corrected P-value < 0.01).

**Transcript Name**	**log2FC**	**p-value**	**Gene Name**	**Linked disease**	**Function**
GSTM1-201 (=) - STRG.5167.1	5.61	0.004	Glutathione S-Transferase Mu 1	Asbestosis and Oral Leukoplakia	Glutathione conjugates formation
BANF1P1-201 (=) - STRG.80167.2	-4.56	0.007	Barrier To Autointegration Factor 1 Pseudogene 1	Leiomyoma	Pseudogene
LRRC37B-211 (o) - STRG.92784.13	-4.13	0.003	Leucine Rich Repeat Containing 37B	Chromosome 17Q11.2 Deletion Syndrome and Chromosome 15Q26-Qter Deletion Syndrome	Protein Coding gene
NR4A3-204 (c) - STRG.57246.1	-3.99	0.005	Nuclear Receptor Subfamily 4 Group A Member 3	Chondrosarcoma, Extraskeletal Myxoid and Chondrosarcoma	May act as a transcriptional activator

Disease Models & Mechanisms • Supplementary information

RNF41-204 (=) - STRG.72097.13	-3.87	0.001	Ring Finger Protein 41	Conversion Disorder and Prader-Willi Syndrome	Encodes for E3 ubiquitin ligase that plays a role in type 1 cytokine receptor signaling
FGF16-201 (=) - STRG.112642.1	3.83	0.007	Fibroblast Growth Factor 16	Metacarpal 4-5 Fusion and Syndactyly, Type Iii	Proper heart development
TNNI3-207 (=) - STRG.103424.2	3.67	0.005	Troponin I3, Cardiac Type	Cardiomyopathy, Dilated, 2A and Cardiomyopathy, Familial Hypertrophic, 7	Inhibitory subunit blocking actin-myosin interactions and thereby mediating striated muscle relaxation
TNNI3-202 (=) - STRG.103424.1	3.63	0.005	Troponin I3, Cardiac Type	Cardiomyopathy, Dilated, 2A and Cardiomyopathy, Familial Hypertrophic, 7	Inhibitory subunit blocking actin-myosin interactions and thereby mediating striated muscle relaxation

Disease Models & Mechanisms • Supplementary information

MCUR1P1-201 (=) - STRG.91073.1	3.43	0.005	MCUR1 Pseudogene 1	N/A	Pseudogene
AP001033.2-201 (=) - STRG.96560.1	3.43	0.005	Long non-coding RNA	N/A	Long non-coding RNA
CHD1-212 (i) - STRG.34005.1	-3.26	0.004	Chromodomain Helicase DNA Binding Protein 1	Pilarowski-Bjornsson Syndrome and Schizophrenia 8	Alters gene expression possibly by modification of chromatin structure
HES3-201 (=) - STRG.307.1	3.18	0.004	Hes Family BHLH Transcription Factor 3	Chromosome 1P36 Deletion Syndrome	Transcriptional repressor of genes that require a bHLH protein for their transcription
MRPL42-210 (=) - STRG.73269.8	-2.93	0.001	Mitochondrial Ribosomal Protein L42	Somatization Disorder	Encodes a protein identified as belonging to both the 28S and the 39S subunits of the mitochondrial ribosome

STRG.21751.25 (u)	2.83	0.006	New intergenic isoform	N/A	N/A
PITPNM2-201 (c) - STRG.74884.1	-2.69	0.009	Phosphatidylinositol Transfer Protein Membrane Associated 2	Retinal Degeneration	Catalyzes the transfer of phosphatidylinositol and phosphatidylcholine between membranes (in vitro). Binds calcium ions.
OSMR-201 (n) - STRG.32126.5	-2.64	0.009	Oncostatin M Receptor	Amyloidosis, Primary Localized Cutaneous, 1 and Primary Cutaneous Amyloidosis	Associates with IL31RA to form the IL31 receptor. Binds IL31 to activate STAT3 and possibly STAT1 and STAT5. Capable of transducing OSM-specific signaling events

Disease Models & Mechanisms • Supplementary information

PRICKLE2-DT-201 (x) - STRG.21751.7	2.44	0.01	Prickle Planar Cell Polarity Protein 2	Sensory Ataxic Neuropathy, Dysarthria, And Ophthalmoparesis and Ataxia Neuropathy Spectrum	Encode for a homolog of Drosophila prickle
AC016542.2-201 (=) - STRG.61654.1	-2.44	0.009	To be Experimentally Confirmed	N/A	N/A
MYO1B-213 (=) - STRG.16666.6	-2.43	0.01	Myosin IB	Colorectal Cancer and Deafness, Autosomal Dominant 48	Motor protein that may participate in process critical to neuronal development and function such as cell migration, neurite outgrowth and vesicular transport
AC004491.1-201 (=) - STRG.46144.21	-2.39	0.005	To be Experimentally Confirmed	N/A	N/A

Disease Models & Mechanisms • Supplementary information

KLHL3-209 (j) - STRG.35076.10	-2.39	0.005	Kelch Like Family Member 3	Pseudohypoaldosteronism, Type Iid and Pseudohypoaldosteronism, Type Iie	Regulator of ion transport in the distal nephron
AC069366.1-201 (=) - STRG.92410.1	-2.39	0.005	Pseudogene	N/A	N/A
NFATC4-201 (=) - STRG.78293.3	2.34	0.007	Nuclear Factor Of Activated T Cells 4	Leukostasis and Trichothiodystrophy 6, Nonphotosensitive	Encoded for a protein that is part of a DNA-binding transcription complex
FGD1-201 (n) - STRG.111935.8	-2.3	0.002	FYVE, RhoGEF And PH Domain Containing 1	Aarskog-Scott Syndrome and Scott Syndrome	Regulates the actin cytoskeleton and cell shape
ZFR2-204 (=) - STRG.99186.1	2.28	0.007	Zinc Finger RNA Binding Protein 2	Malignant Essential Hypertension	Zinc Finger RNA Binding Protein

Disease Models & Mechanisms • Supplementary information

ASAP2-201 (n) - STRG.10409.8	-2.22	0.002	ArfGAP With SH3 Domain, Ankyrin Repeat And PH Domain 2	Bulbar Polio	Activates the small GTPases ARF1, ARF5 and ARF6. Regulates the formation of post-Golgi vesicles and modulates constitutive secretion. Modulates phagocytosis mediated by Fc gamma receptor and ARF6. Modulates PXN recruitment to focal contacts and cell migration
SAR1B-206 (=) - STRG.34986.7	-2.19	0.006	Secretion Associated Ras Related GTPase 1B	Chylomicron Retention Disease and Hypobetalipoproteinemia, Familial, 1	Involved in transport from the endoplasmic reticulum to the Golgi apparatus.
Y_RNA.110-201 (=) - STRG.65888.19	-2.18	0.003	miscellaneous RNA	N/A	Miscellaneous small RNA

Disease Models & Mechanisms • Supplementary information

ZFR2-201 (j) - STRG.99186.2	2.13	0.007	Zinc Finger RNA Binding Protein 2	Malignant Essential Hypertension	Zinc Finger RNA Binding Protein
FNBP4-205 (c) - STRG.65888.2	-2.11	0.004	Formin Binding Protein 4	Microphthalmia With Limb Anomalies and Cerebral Amyloid Angiopathy, Itm2b-Related, 2	Regulation of cytoskeletal dynamics during cell division and migration and maintenance of membrane curvature at sites of nascent vesicle formation
PPM1N-206 (=) - STRG.102456.3	2.07	0.004	Protein Phosphatase, Mg2+/Mn2+ Dependent 1N (Putative)	N/A	Protein Phosphatase

Disease Models & Mechanisms • Supplementary information

KRT7-201 (=) - STRG.71856.1	2.06	0.002	Keratin 7	Pseudomyxoma Peritonei and Signet Ring Cell Adenocarcinoma	Blocks interferon-dependent interphase and stimulates DNA synthesis in cells. Involved in the translational regulation of the human papillomavirus type 16 E7 mRNA (HPV16 E7)
CSNK1G1-209 (o) - STRG.84144.5	-2.01	0.002	Casein Kinase 1 Gamma 1	Aortic Valve Prolapse and Gm1-Gangliosidosis, Type I	Cell cycle checkpoint arrest in response to stalled replication forks by phosphorylating Claspin
AL391005.1-201 (=) - STRG.64011.1	1.96	0.009	To be Experimentally Confirmed	N/A	N/A

Disease Models & Mechanisms • Supplementary information

MTURN-201 (j) - STRG.44254.3	-1.96	0.007	Maturin, Neural Progenitor Differentiation Regulator Homolog	Polycystic Kidney Disease 2 With Or Without Polycystic Liver Disease	Promotes megakaryocyte differentiation and represses NF-kappa-B transcriptional activity
LRRC37B-204 (j) - STRG.92784.12	-1.92	0.006	Leucine Rich Repeat Containing 37B	Chromosome 17Q11.2 Deletion Syndrome and Chromosome 15Q26-Qter Deletion Syndrome	Protein Coding gene
POLD1-212 (i) - STRG.103029.2	1.92	0.005	DNA Polymerase Delta 1, Catalytic Subunit	Mandibular Hypoplasia, Deafness, Progeroid Features, And Lipodystrophy Syndrome and Colorectal Cancer 10	Role in DNA replication and repair

Disease Models & Mechanisms • Supplementary information

ARSI-201 (=) - STRG.35725.1	1.89	0.006	Arylsulfatase Family Member I	Autosomal Recessive Spastic Paraplegia Type 66 and Gastric Dilatation	Protein encoded by this gene is thought to be secreted, and to function in extracellular space
LINC02334-202 (j) - STRG.76096.1	-1.85	0.004	Long Intergenic Non-Protein Coding RNA 2334	N/A	Long non-coding RNA
IFITM1-202 (=) - STRG.64176.1	1.8	0.005	Interferon Induced Transmembrane Protein 1	Influenza and West Nile Virus	Restricts cellular entry by diverse viral pathogens, such as influenza A virus, Ebola virus and Sars-CoV-2

Disease Models & Mechanisms • Supplementary information

Table S2. GO enrichment analysis results.

Most differentially expressed transcripts uncovered by long read single molecule cDNA sequencing (Oxford Nanopore) revealed an enrichment of the transcription coactivator activity function with 8 genes involved in this function found differentially expressed (genes coding for transcripts with batch-corrected P-value < 0.01 and |log2(FoldChange)|>0.5 analyzed with GOrilla from Gene Ontology (Eden et al., 2009)).

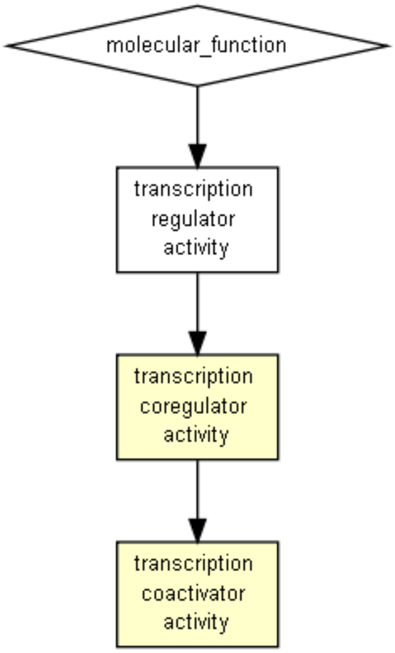

GO term	Description	P-value	FDR q-value	Enrichment (N, B, n, b)	Genes
GO:0003713	transcription coactivator activity	6.93E-5	6.62E-2	3.87 (348,8,90,8)	HCFC1 SMARCC1 BCL9L SMARCA4 MED23 DDX17 NCOA1 MTA3
GO:0003712	transcription coregulator activity	8.55E-4	4.08E-1	2.97 (348,13,90,10)	HCFC1 SMARCC1 BCL9L SMARCA4 MED23 DDX17 NSD1 BTG1 NCOA1 MTA3

Disease Models & Mechanisms • Supplementary information

Table S3. List of Taqman gene expression assays for qPCR analysis

Gene name	Source	Identifier
CXCR4	Thermo Fisher Scientific	Hs00607978_s1
GATA4	Thermo Fisher Scientific	Hs00171403_m1
SOX17	Thermo Fisher Scientific	Hs00751752_s1
SOX2	Thermo Fisher Scientific	Hs04234836_s1
PAX9	Thermo Fisher Scientific	Hs00196354_m1
ISL1	Thermo Fisher Scientific	Hs00158126_m1
NKX2.1	Thermo Fisher Scientific	Hs00968940_m1
KRT4	Thermo Fisher Scientific	Hs00361611_m1
KRT13	Thermo Fisher Scientific	Hs02558881_s1
P63	Thermo Fisher Scientific	Hs00978340_m1
INV	Thermo Fisher Scientific	Hs00846307_s1

Table S4. List of primary and secondary antibodies

Antibodies	Source	Dilution	Identifier
Mouse monoclonal antibody to TFF1	Abcam	1:50	ab72876
Rabbit monoclonal antibody to CXCR4	Abcam	1:200	ab181020
Mouse monoclonal antibody to SOX2	Abcam	1:250	ab79351
Rat monoclonal antibody to PAX9	Abcam	1:100	ab28538
Rabbit monoclonal antibody to cytokeratin 4	Abcam	1:50	ab51599
Mouse monoclonal antibody to SOX17	Abcam	1:100	ab84990
Rabbit monoclonal antibody to ISL1	Abcam	1:100	ab109517
Rabbit polyclonal antibody to GATA4	Thermo Fisher Scientific	1:50	PA1-102
Mouse monoclonal antibody to INV	Abcam	1:100	ab68
Rabbit polyclonal antibody to P63	Abcam	1:200	ab53039
Rabbit monoclonal antibody to KRT13	Abcam	1:100	ab92551
Rabbit polyclonal antibody to AFP (ready to use)	DAKO Omnis	N/A	GA50061-2
**Secondary antibodies**			
Goat anti-mouse IgG H&L (Alexa Fluor 488)	Abcam	1:1000	ab150113
Goat anti-mouse IgG H&L (Alexa Fluor 594)	Abcam	1:1000	ab150120
Donkey anti-rabbit IgG H&L (Alexa Fluor 488)	Thermo Fisher Scientific	1:1000	A21206
Goat anti-rabbit IgG H&L (Alexa Fluor 488)	Abcam	1:1000	ab150077

Disease Models & Mechanisms • Supplementary information

Table S5. Generated DE cells in both groups showed high Ct values by qPCR (39-40). We did not have a human positive control cell line expressing OTX2 such as the brain tissue to confirm the ectodermal commitment in both groups in order to calculate DDCt values. Therefore, we relied on the high CT values to conclude that OTX2 was absent.

**H9 DE**	*Ct values*
GAPDH	18.91
OTX2	40
**iPSC DE**	
GAPDH	19.25
OTX2	39.38
**Patient 1 DE**	
GAPDH	19.64
OTX2	40
**Patient 2 DE**	
GAPDH	20.5
OTX2	40
**Patient 3 DE**	
GAPDH	19.9
OTX2	40
